# Structural and Dynamical Effects of the CaO/SrO Substitution in Bioactive Glasses

**DOI:** 10.3390/molecules29194720

**Published:** 2024-10-05

**Authors:** Margit Fabian, Matthew Krzystyniak, Atul Khanna, Zsolt Kovacs

**Affiliations:** 1 HUN-REN Centre for Energy Research, Konkoly Thege Miklos st. 29-33, 1121 Budapest, Hungary; fabian.margit@ek.hun-ren.hu; 2 ISIS Facility, Rutherford Appleton Laboratory, Chilton, Didcot OX11 0QX, UK; 3 Department of Physics, Guru Nanak Dev University, Amritsar 143005, India; atul.phy@gndu.ac.in; 4 Department of Materials Physics, Institute of Physics, Eötvös Loránd University, Pázmány Péter st. 1/a, H-1117 Budapest, Hungary; kovacs.zsolt@ttk.elte.hu

**Keywords:** bioactive glasses, isotopic disorder, force-constant disorder, nuclear momentum distribution, neutron diffraction, neutron Compton scattering, reverse Monte Carlo modelling, Raman spectroscopy

## Abstract

Silicate glasses containing silicon, sodium, phosphorous, and calcium have the ability to promote bone regeneration and biodegrade as new tissue is generated. Recently, it has been suggested that adding SrO can benefit tissue growth and silicate glass dissolution. Motivated by these recent developments, the effect of SrO/CaO–CaO/SrO substitution on the local structure and dynamics of Si-Na-P-Ca-O oxide glasses has been studied in this work. Differential thermal analysis has been performed to determine the thermal stability of the glasses after the addition of strontium. The local structure has been studied by neutron diffraction augmented by Reverse Monte Carlo simulation, and the local dynamics by neutron Compton scattering and Raman spectroscopy. Differential thermal analysis has shown that SrO-containing glasses have lower glass transition, melting, and crystallisation temperatures. Moreover, the addition of the Sr^2+^ ions decreased the thermal stability of the glass structure. The total neutron diffraction augmented by the RMC simulation revealed that Sr played a similar role as Ca in the glass structure when substituted on a molar basis. The bond length and the coordination number distributions of the network modifiers and network formers did not change when SrO (x = 0.125, 0.25) was substituted for CaO (25-x). However, the network connectivity increased in glass with 12.5 mol% CaO due to the increased length of the Si-O-Si interconnected chain. The analysis of Raman spectra revealed that substituting CaO with SrO in the glass structure dramatically enhances the intensity of the high-frequency band of 1110–2000 cm^−1^. For all glasses under investigation, the changes in the relative intensities of Raman bands and the distributions of the bond lengths and coordination numbers upon the SrO substitution were correlated with the values of the widths of nuclear momentum distributions of Si, Na, P, Ca, O, and Sr. The widths of nuclear momentum distributions were observed to soften compared to the values observed and simulated in their parent metal-oxide crystals. The widths of nuclear momentum distributions, obtained from fitting the experimental data to neutron Compton spectra, were related to the amount of disorder of effective force constants acting on individual atomic species in the glasses.

## 1. Introduction

The demand for bone implants has risen significantly in recent years. Severely injured bone tissue may require treatment involving bone implantation or transplantation. Despite much study and effort, a viable implant alternative for bone tissue regeneration has remained elusive. One of the prerequisites for developing successful implant materials is to design a model that incorporates non-toxic, biodegradable materials that can give an architectural approach for cells to attach, grow, and generate an extracellular matrix.

The first bioactive glass was discovered by Hench in 1969, who found that certain compositions of SiO_2_-P_2_O_5_-CaO-Na_2_O glasses could form a strong interfacial bond between the glass and the host tissue [1]. Since then, the field of bioactive ceramics has evolved to produce many bioactive glasses and glass ceramics, such as synthetic hydroxyapatite (HA) and calcium phosphates [2]. Nowadays, the most common and often-used bioactive glass in clinical applications is 45S5 Bioglass^®^ [3,4]. Despite the outstanding biocompatibility and bioactivity of bioactive glasses, their commercial success has been low, owing to the fact that clinical applications have so far been limited to particulate bioactive glass types, only suitable for non-load-bearing sites [5].

Recent research aiming at further commercialising bioactive glasses has concentrated on substituting strontium in the glass structure. It has been discovered that, besides Na and Pb, Sr can be substituted in the Ca positions of apatite [6]. The basic properties that distinguish these glasses from conventional Na-Ca-Si products are a relatively low content of silica, less than 60 mol%, and a relatively high Na_2_O and CaO content. These peculiarities in the glass formulation confer a high reactivity in aqueous media. Sr ions, improving osteoblast activity and inhibiting osteoclast function, can enhance the density of the bone tissue, resulting in a significant reduction in fracture risk. Once it is incorporated into the bone, strontium acts similarly to calcium but with a slight difference regarding intestinal absorption, renal excretion, and bone accumulation [7]. In vivo studies suggest other beneficial effects of strontium substitution, such as anti-inflammatory and bone growth enhancement [8,9]. Strontium ranelate (Protelos^®^) [10,11] has been successfully applied as an anti-osteoporosis drug. It is known to promote bone-forming osteoblast cells and inhibit bone-resorbing osteoclast cells [9,12].

Lao et al. studied the in vitro reactivity of the SiO_2_-CaO-SrO and SiO_2_-CaO-P_2_O_5_-SrO bioactive glasses [13]. They suggested that Sr-containing materials had a lower dissolution rate than that of Sr-free materials but a better transformation rate of the HA on the surface of the glass. The substitution of heavier Sr atoms indirectly increased the glass silica content. Consequently, it led to an increase in network connectivity and a decrease in glass dissolution [13]. In the study of Gentleman et al., Sr was substituted for the Ca in the bioactive glass SiO_2_-P_2_O_5_-CaO-Na_2_O [8]. The substitution caused an ion release from the bioactive glass, enhancing metabolic bioactivity in the osteoblast cells. Moreover, it was suggested that it may have a similar effect as the anti-osteoporosis drug strontium ranelate in vivo [8]. The current studies indicate that since network connectivity directly affects bioactivity, there should be careful consideration regarding the strontium substitution on a molar basis [9].

Motivated by these recent developments, our study focuses on elucidating the local structure and dynamics of Sr-containing bioactive oxide glasses with different Sr/Ca ratios. Our main goal is to understand the effects of SrO/CaO–CaO/SrO substitution on the short-range (defined in terms of structural units) and intermediate-range order (in terms of the connectivity between structural units), as well as changes in the local (occurring within the range of typical atomic displacements) glassy dynamics, on the durability and its antibacterial properties of the glass systems. To this end, we have conducted Differential Thermal Analysis (DTA) to determine the thermal property changes upon the SrO incorporation. Moreover, the structural analysis of the glasses was carried out using neutron diffraction measurements supported by the Reverse Monte Carlo (RMC) simulation. Diffraction experiments provide the most suitable tool to get information on the atomic configuration in terms of first neighbour distances and coordination numbers. Apart from the local structural information inferred from diffraction studies augmented with RMC, functional disordered materials need local investigation of dynamical properties and, most crucially, the interatomic force constants. Force-constant values and their distributions in disordered systems are believed to be responsible for a plethora of mechanical and thermodynamic properties. However, only a few experimental techniques can be applied to reveal the force-constant distributions in glasses. Raman spectroscopy can give information about the changes in the formation of silicate and phosphorus units upon the replacement of CaO with SrO ^®^ [4,14,15]. Apart from Raman spectroscopy, in this work, we apply one of the hitherto largely unexplored techniques for the assessment of the magnitudes of the local force constants and the force-constant disorder in glassy systems, neutron Compton scattering (NCS) [16,17,18,19,20]. The application of the NCS technique allows us to analyse the disorder-induced softening of force constant and the force-constant distributions in an isotope-resolved manner. The isotopic mass character of the NCS is due to the sensitivity of the nuclear momentum distributions underlying the shapes of the recoil peaks present in the NCS spectra to the local isotope-projected vibrational densities of states. In this respect, the NCS is complementary to dynamical probes such as Raman spectroscopy, and the concurrent application of both techniques, along with neutron diffraction and DTA, paints a global picture of local structure–dynamics correlations in this important class of functional materials.

## 2. Results 

### 2.1. Thermal Analysis of the Bioactive Glass Samples

**Figure 1:**
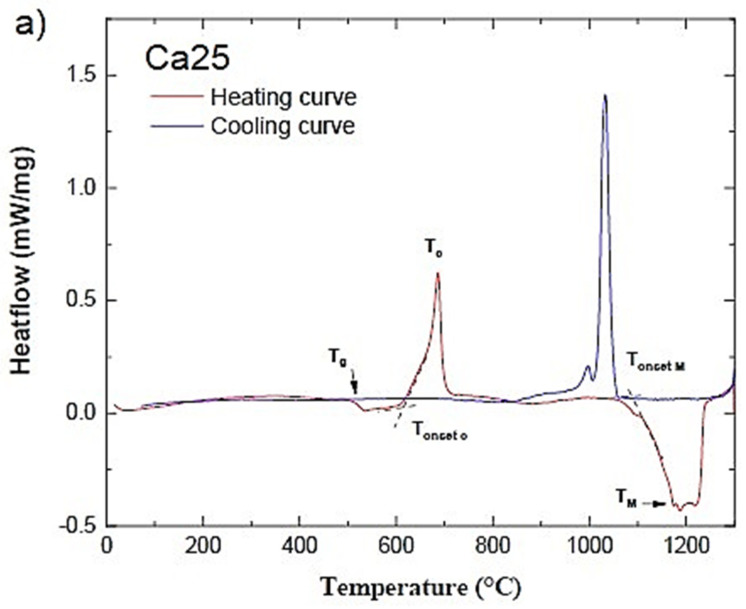

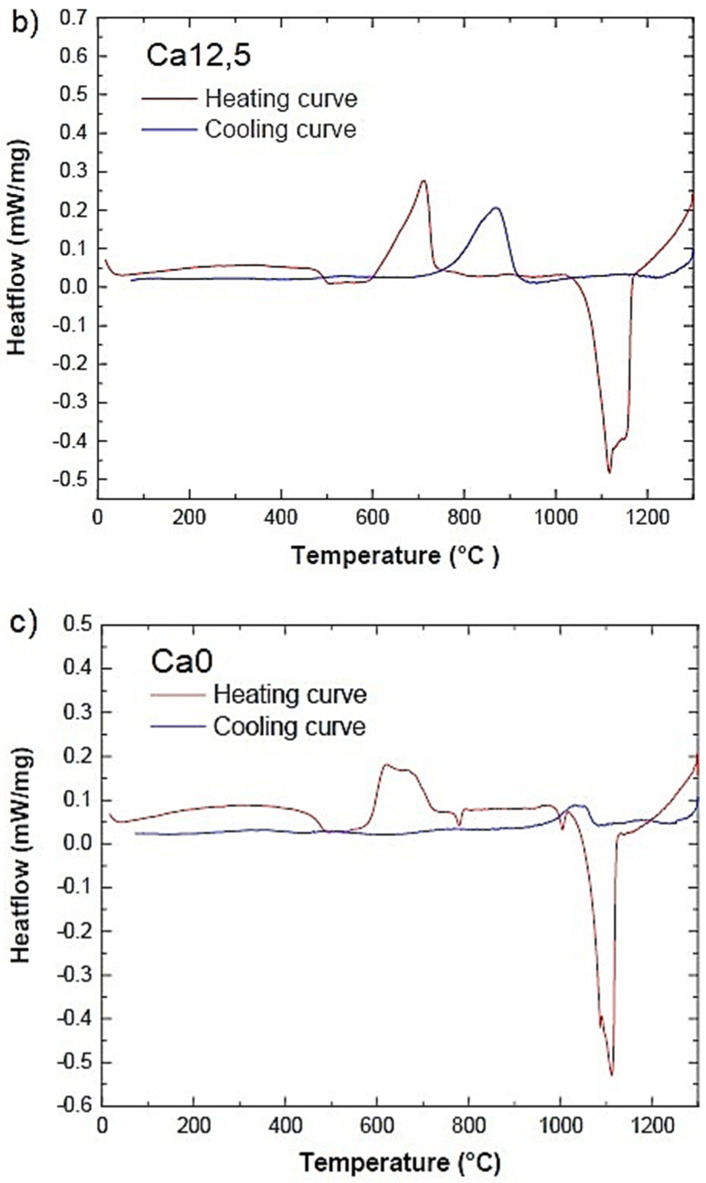
DTA curves of Ca25 (**a**), Ca12.5 (**b**), and Ca0 (**c**) glassy samples. See text for details.

In Figure 1, the DTA curves of the glass samples are shown. The DTA traces of the first sample (see Figure 1a) with a composition of 0% SrO exhibit an endothermic effect at 508 °C caused by the glass transition, followed by an exothermic peak of the crystallisation beginning at 615 °C. Melting takes place between 1112 and 1189 °C.

Similarly, for all the samples, the glass transition temperature was identified at the inflexion point at the beginning of the first endotherm regime during heating. The crystallisation onset (T_onset c_) and peak temperatures (T_c_) were found at the beginning and maximum of the exothermic peak, respectively. Typically, a broad crystallisation peak is associated with surface crystallisation, whereas a sharp peak is associated with bulk crystallisation [21]. It is well known that the higher the T_onset c_-T_g_ value, the more nucleation and crystallisation processes are inhibited, and the glass becomes more stable [22]. The onset temperature of the endothermic melting process (T_onset M_) was also determined from the heating curves. Cooling curves show the same phase transformation (i.e., solidification) starting at or slightly below T_onset M_. The glass transition temperature (T_g_) and the other characteristic temperatures are summarised in Table 1 for all glass compositions. A decrease in T_g_ from 508 °C to 455 °C when SrO was substituted with CaO indicates the decrease in stability of the glass network when Sr^2+^ ions are introduced into the glass. The melting temperature (T_onset M_) also decreased with increasing SrO content, whilst T_onset c_–T_g_, the temperature difference between the glass transition and the onset of crystallisation, increases with the addition of Sr. The smaller the difference, the easier it is for the Sr-free composition to crystallise. The decrease in the value of this temperature difference from 134 to 104 °C (Table 1) indicates that glass stability for crystallisation decreases with the substitution of CaO by SrO. Similarly, when the amount of strontium oxide in the bioactive glass increases, the latent heat value of the glass drops.

**Figure 2:**
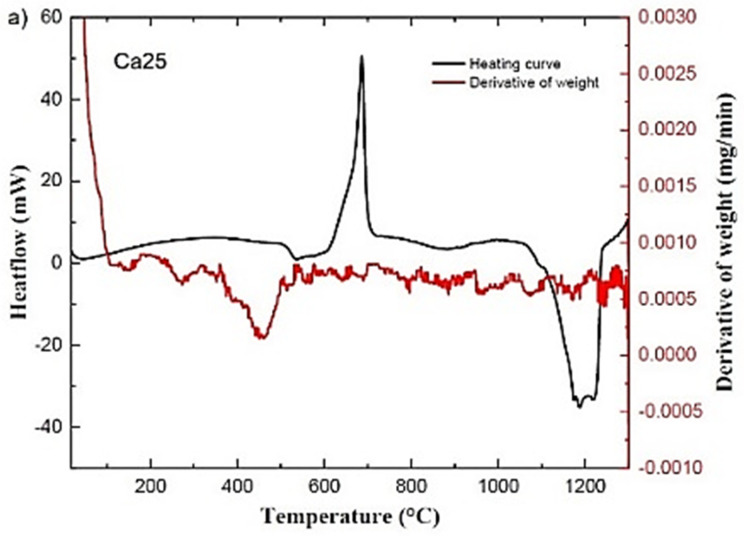

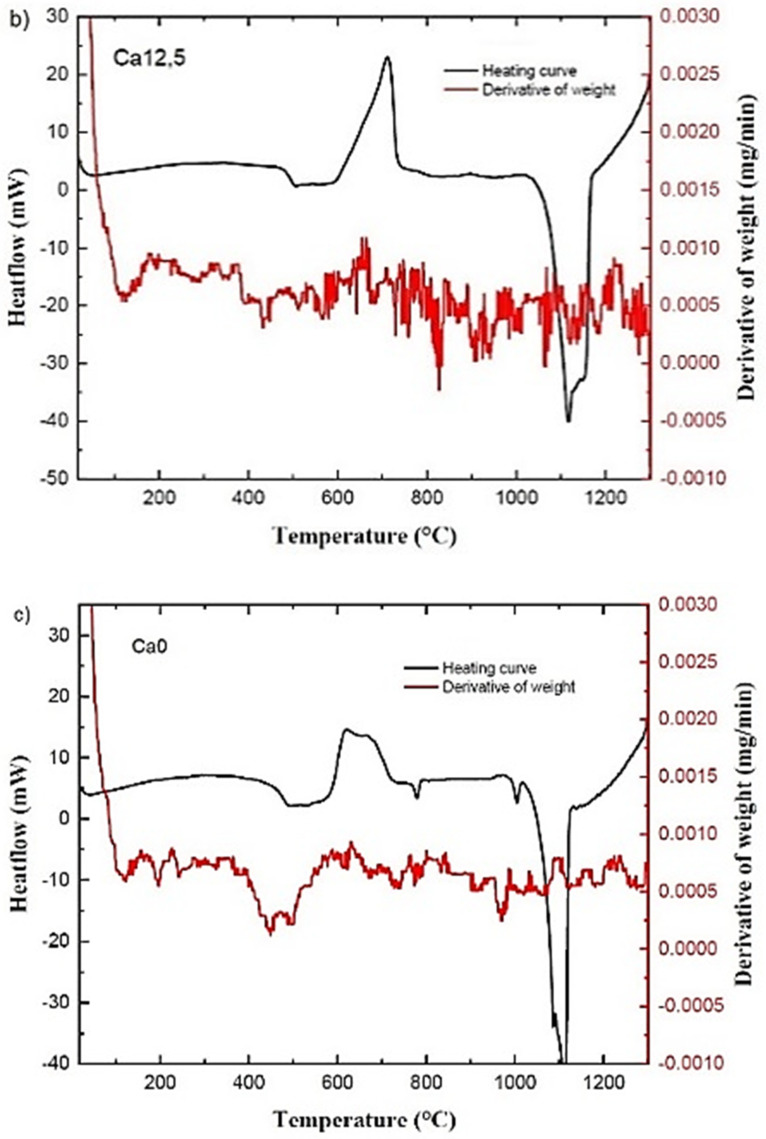
DTA and DTG curves of Ca25 (**a**), Ca12.5 (**b**), and Ca0 (**c**) glassy samples. See text for details.

Figure 2 shows the DTA and DTG curves of the glass samples. Weight loss before nucleation is apparent in the DTG and DTA curves as a function of temperature. In the case of the Ca25 sample, it is between 375–517 °C, whilst for the Ca0 sample, it is in the 398–557 °C range.

### 2.2. Local Structure from Neutron Diffraction

**Figure 3:**
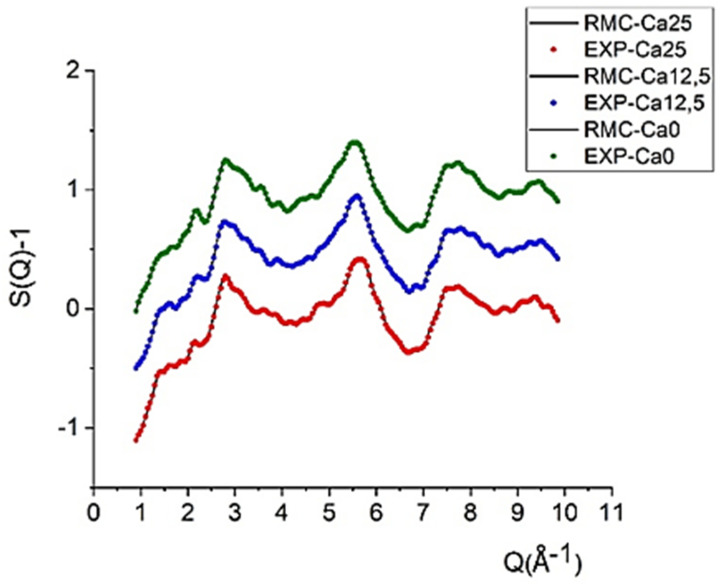
Experimental and RMC calculated structure factors of Ca25 (red), Ca 12.5(blue), and Ca0 (green) glass samples. Curves are displaced by 1 unit successively for clarity. See text for details.

**Figure 4:**
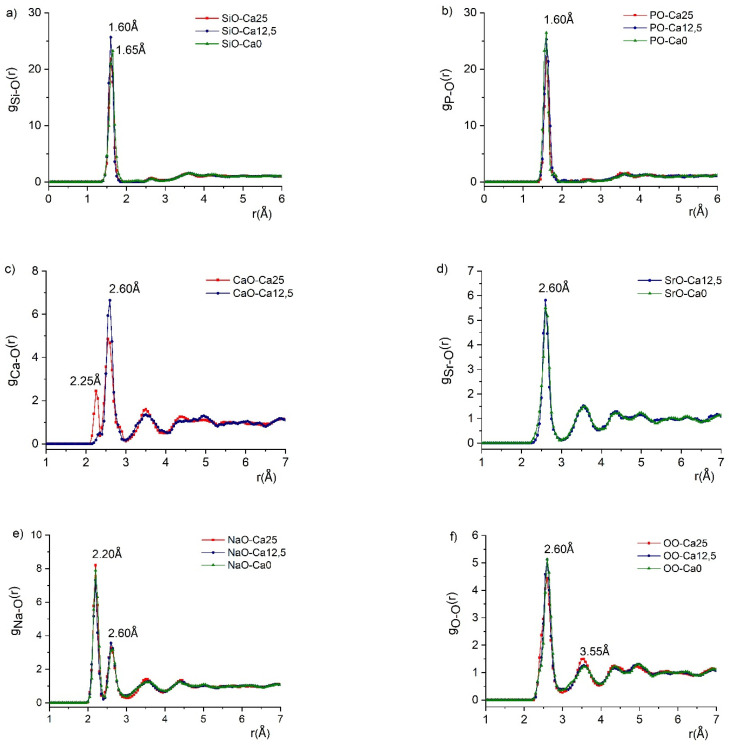
Partial atomic pair correlations for Si-O (**a**), P-O (**b**), Ca-O (**c**), Sr-O (**d**), Na-O (**e**), and O-O (**f**), in glassy samples (Ca25 (red), Ca12.5 (blue), Ca0 (green)). The peak positions corresponding to key bond lengths are shown. See text for details.

**Figure 5:**
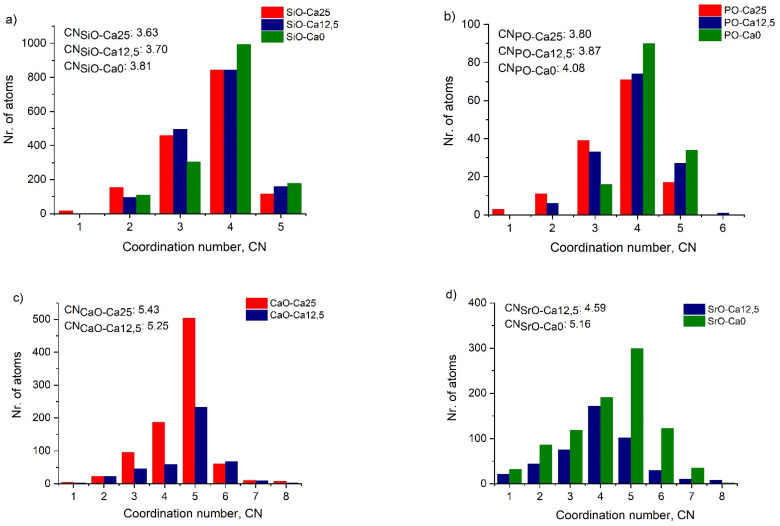
Si-O (**a**), P-O (**b**), CaO (**c**), and O-O (**d**) coordination number distributions in the glassy samples.

The results of the RMC analysis in terms of experimental and RMC calculated structure factors, partial atomic pair correlation function and coordination number distributions are shown in Figure 3, Figure 4 and Figure 5, respectively. The RMC-simulated structure factors agree very well with the experimental ones for all three samples, Ca0, Ca12.5, and Ca25, as shown in Figure 3. At long distances (small Q values), the main features of the S(Q) curves are similar for all three samples. However, at short distances (large Q values), some differences manifest as a function of the Ca/Sr ratio.

From the RMC simulation, several well-converged partial pair-correlation functions, g_ij_(r), and coordination number distributions, CN_ij_, were obtained (Figure 4). Si-O covalent bond length of 1.60 ± 0.01 Å was obtained for the Ca25 and Ca12.5 samples (Figure 4a), showing a good agreement with values published in the literature for this network former oxide [23,24].

For the Ca0 sample, the bond length value was 1.65 ± 0.02 Å (Figure 4b). The average coordination number distribution (Figure 5) shows that four oxygen atoms surround Si atoms, but the number of 4-fold coordination units depends on the concentration, increasing with decreasing calcium concentration. The average Si-O coordination numbers are 3.63 ± 0.05, 3.70 ± 0.05, and 3.81 ± 0.05 for the Ca25, Ca12.5, and Ca0, respectively.

The P-O first neighbour distance shows a characteristic peak at 1.60 ± 0.01 Å for all three samples (Figure 4b). RMC calculations have revealed that the P atoms are mainly 4-fold coordinated by oxygen atoms (Figure 5b), and this number increases slightly with decreasing CaO concentration from 25 mol% to 0 mol%. This result is in excellent agreement with data reported in our previous work [23] and the literature [25,26,27].

All investigated samples contain similar Si/P concentration ratios, with their glassy network containing both SiO_4_ and PO_4_ units. The Ca-O first neighbour distributions exhibit characteristic peaks at 2.60 ± 0.02 Å for all samples (Figure 4c). Additionally, the Ca25 sample has a shoulder peak at 2.25 Å in the distribution. The coordination number distribution analysis has revealed that the Ca atoms are 5-fold coordinated in both glasses (Figure 5c).

CaO plays two important roles in bioactive glasses. Firstly, it is expected to improve the bioactivity of the glasses. Secondly, it serves as a modifier in the glass structure. Some of the previous studies found shorter CaO distances at 2.43 Å [28] and 2.35 Å [29]. However, the study of a sample with a similar composition (SiO_2_-Na_2_O-CaO-SrO) showed a double peak for CaO distances at 2.25 Å and 2.65 Å, and a coordination number distribution peaking at 5, for the Ca atom coordinated by five oxygen atoms [30], with both types of results agreeing well with our findings.

The Sr-O distance distribution shows an intensive peak at 2.60 ± 0.01 Å in both strontium-containing samples (Figure 4d), in agreement with the literature [30,31]. Even with increasing Sr concentration, the peak position and intensity do not change. The coordination number of Sr with oxygen atom is 4.59 ± 0.05 in the Ca12.5 sample (Figure 5d). It increases to 5.16 ± 0.05 for the Ca0 sample, a value smaller than 6, which is typically found in the literature corresponding to SrO_6_ units.

The Na-O correlation functions consist of double peaks with characteristic distances at 2.20 ± 0.02 Å and 2.60 ± 0.02 Å (Figure 4e). The O-O distribution (Figure 4f) shows two peaks at 2.60 ± 0.03 and 3.55 ± 0.05 Å. The O-O average coordination number is close to 5.

The fact that the structure factors are similar for the Si-Ca and Sr-Na-P-O glasses indicates that no significant structural changes occur as Ca is replaced by Sr. The main building blocks in the structures of the glass samples are the SiO_4_ and PO_4_ units. In the 45SiO_2_-12.5CaO-28Na_2_O-2P_2_O_5_-12.5SrO sample, half of the initial amount of Ca is substituted by the Sr, the Ca^2+^ cations being substituted by Sr^2+^. Ca and Sr are alkaline-earth elements and act as network modifiers in the silicate structure. However, the bond length and the coordination number distributions of Ca and Sr cations in all considered samples show some interesting differences. In the case of the Ca12.5 sample, both Ca-O and Sr-O coordination numbers are lower than expected, even though the bond length shows the main peak at the same distance, 2.60 Å. Whilst the incorporation of Sr content lowers the coordination number of the network modifiers, the network formers show little to no change.

### 2.3. Raman Analysis

**Figure 6:**
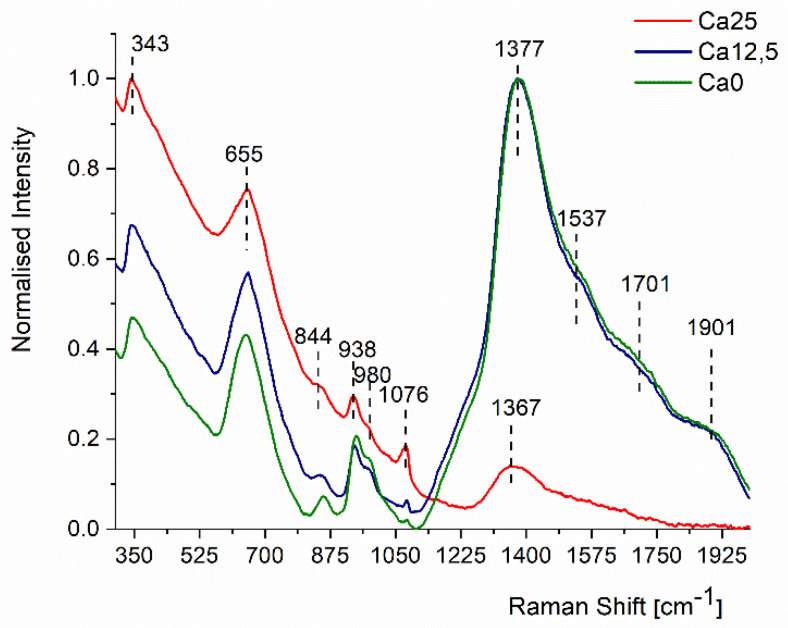
Effects of replacement of CaO with SrO on the Raman spectra of phosphosilicate glasses. See text for details.

Figure 6 displays the intensity normalised Raman spectra of three glass samples in the phonon wavenumber range of 300–2000 cm^−1^. All the spectra show three main vibrational bands: 300–810 cm^−1^, 810–1100 cm^−1^, and the high-frequency band 1110–2000 cm^−1^.

The substitution of CaO with SrO in the glass structure dramatically enhances the relative intensity of the high-frequency band of 1110–2000 cm^−1^. In the sample containing 25 mol% CaO, the low-frequency bands of 300–810 cm^−1^ have higher relative intensity than that of the high-frequency Raman band, an effect earlier reported in the literature [32]. The bands in the 300–810 cm^−1^ range are due to the superposition of Si-O-Si, P-O-P, and Si-O-P bending vibration modes [32,33]. The multiple peaks in the 810–1110 cm^−1^ range are due to Si-O and P-O stretching vibration modes [32,33]. The high-frequency band centered at 1367–1377 cm^−1^ is due to P=O stretching vibrations [33].

This high-frequency band is relatively weak in the sample containing 25 mol% CaO. However, its intensity increases dramatically by the partial and complete replacement of CaO with SrO in the phosphosilicate glass network. The mid-frequency band at 844 cm^−1^ shows only a weak shoulder in the first sample that contains only Ca^2+^ ions. On replacing 12.5 mol% of CaO with 12.5 mol% of SrO, this band becomes more pronounced and transforms into a well-resolved peak and shifts to a higher frequency of 856 cm^−1^ on replacing all the Ca^2+^ with Sr^2+^. Furthermore, the peak at 1076 cm^−1^ becomes significantly suppressed when Sr^2+^ is added to the glass network, indicating that this band (at 1076 cm^−1^) is due to the Si-O stretching modes. Thus, the P-O and P=O stretching vibrations become increasingly stronger with the substitution of CaO by SrO, and the enhanced bioactivity of Sr-containing phosphosilicate glasses can be explained by more active vibration modes of phosphate structural groups [34].

### 2.4. Neutron Compton Scattering Results

**Figure 7:**
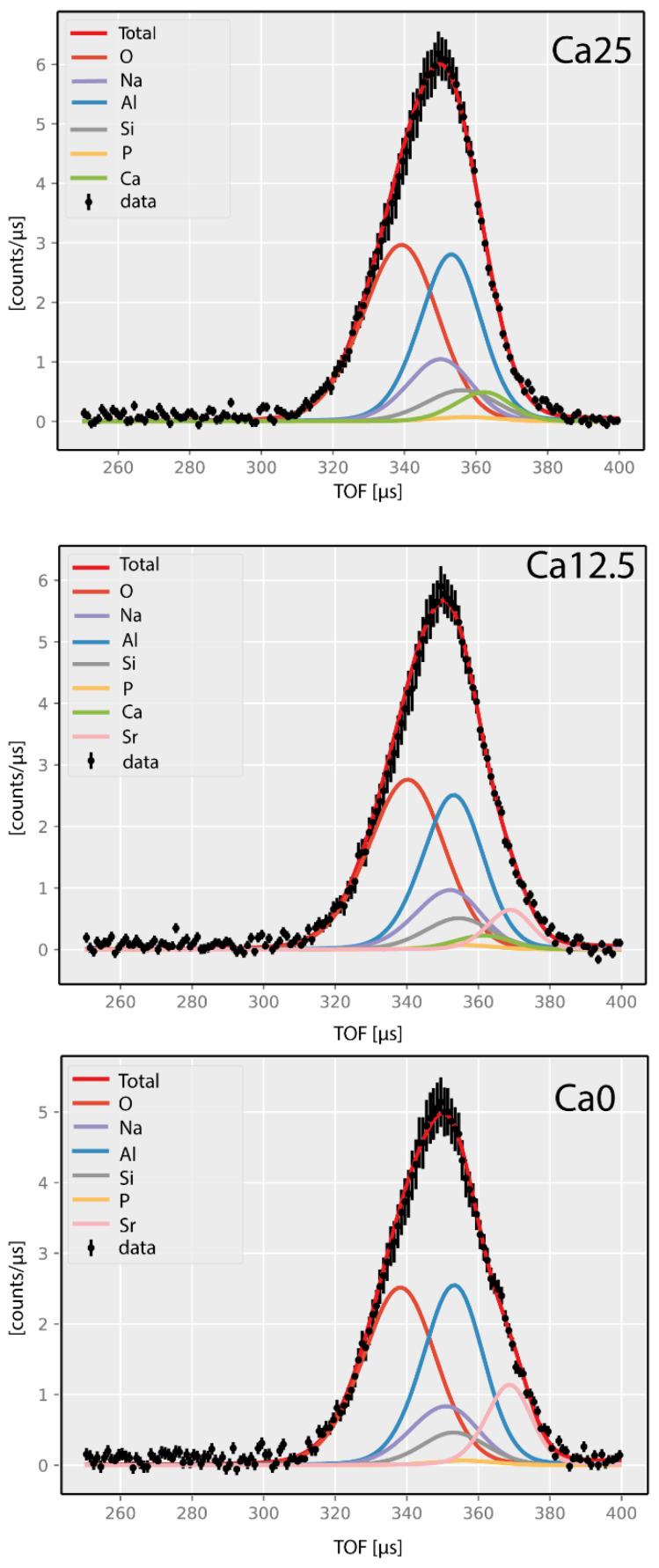
Fits of the TOF spectra recorded at VESUVIO for bioactive glasses Ca25, Ca12.5, and Ca0. Recoil peaks of individual atomic species in the glasses have been colour-coded, with the recoil peaks due to the aluminium container marked in blue. See text for details.

Figure 7 shows the mass-resolved NCS spectra of the three glasses under consideration. Due to the impulsive nature of neutron Compton scattering, each recoiling atomic mass produces its recoil peak in the time-of-flight (TOF) domain due to the distribution of its momenta in an underlying local potential of the mean force [16,20]. In the momentum space of each nucleus, the spread of its momentum distribution is always proportional to the mass of the nucleus. However, this trend reverses in the TOF domain, and the lightweight nuclei produce broader recoil peaks [16,20]. Moreover, since VESUVIO is an inverse-geometry spectrometer, the order in which the recoil peaks appear in the TOF domain is from the lowest recoiling mass at the smallest possible values of the TOF towards heavier recoiling masses at the highest TOF values [16,20].

When data summed over all scattering detectors are shown, as the scattering angles grow, the positions of recoil peaks for individual masses change, and, for a given fixed mass, the recoil peak at a low scattering angle appears at larger TOF values than its counterpart recorded by a detector placed at a higher scattering angle [16,20]. However, the lower the atomic mass value, the more the recoil peak positions of this mass are sensitive to the change in the scattering angle (detector) [16,20]. Thus, for instance, the oxygen recoil peaks, shown in Figure 7 as solid red lines, spread between 300 and 370 microseconds as a result of the combination of two factors: (i) the oxygen recoil peak positions moving from left to right as the scattering angles change from ca. 170 to ca. 130 degrees, and (ii) oxygen momentum distributions for each recoil peak spreading over finite regions in the TOF domain. Furthermore, the recoil peaks of aluminium sample containers, shown in Figure 6 and Figure 7 as solid blue lines, occupy the regions between ca 330 and 380 microseconds. The TOF region between ca 320 and 380 microseconds is filled by the recoil peaks of Na, Si, P, K, Ca, and Sr, with the peaks progressing to increasing atomic masses.

The integral intensities of the recoiling peaks of different atomic masses recorded in the TOF domain depend on the sample stoichiometry and the total bound scattering neutron cross-section values of individual atomic species [16,20]. Thus, knowing each sample stoichiometry and the values of the tabulated total bound scattering neutron cross-sections, one can constrain the ratios of peak area in fitting the NCS spectra. This procedure, known as stoichiometric fixing, applied successfully in fitting NCS spectra of solid-state systems, including glasses [35], has also been used in this work and resulted in good-quality fits, shown in Figure 6 as solid red lines. It is worth noting that, in Figure 7, sums of spectra and sums of fits to individual spectra recorded by individual detectors in backscattering on VESUVIO are shown. The data recorded for individual detectors were fitted sequentially first, and the values of peak widths and peak intensities obtained from fitting individual detector data were then averaged over the entire detector group and compared to theoretical predictions. This commonly used data treatment protocol has been successfully applied in fitting NCS spectra of solid-state systems and molecules [18,35].

Following the methodology presented in our previous work on molybdate glasses and their parent metal oxides [18], the values of the standard deviations of the nuclear momentum distributions of individual atomic species present in the Ca12.5, Ca25, and Ca0 samples were classified following the methodology presented in our previous work on molybdate glasses and their parent metal oxides by placing them between two extremes: (i) the values obtained from the Maxwell–Boltzmann distribution of completely non-interacting nuclei in the absence of any binding potential (restoring force), and (ii) the values obtained from the harmonic lattice dynamics DFT simulations conducted under periodic boundary conditions representing completely ordered atomic species located in crystal lattices of the parent metal oxides.

The computational strategy can be summarised as follows:

(i) Assume that the disorder in glasses will always act towards the softening of individual vibrational modes, and thus the softening of the atom-projected vibrational densities of states (apVDOSs) of individual atomic species, as measured with respect to the apVDOSs obtained by the simulation of the perfectly periodic atomic structures of the parent metal oxides. For instance, the silicon and oxygen atoms in a glassy SiO_2_ will always have their apVDOSs softened with respect to their counterparts in the polycrystalline SiO_2_. In the case of oxygen atomic species present in a glassy system whose composition is a mixture of different metal oxides, for the comparison, provide a weighted average of the simulated oxygen-projected VDOSs of different metal oxides.
(ii) Assume that, for any type of atomic species bound in a solid/glass system, the values of the standard deviation of the nuclear momentum distribution (NMD width) can be bound from below by the value obtained from the Maxwell–Boltzmann distribution (MBD) in the absence of any binding potential (for a completely free and non-interacting atomic particle) that depends only on the temperature and mass of the atomic species under consideration.
(iii) Simulate the apVDOSs of the parent metal oxides and obtain the values of the standard deviations (NMD widths) of nuclear momentum distributions as Boltzmann population factor-weighted centres of gravity of the apVDOSs (according to Equation (3)).
(iv) Compare the simulated values of the NMD widths with their counterparts obtained by fitting the experimental momentum distributions (assuming their Gaussian shapes).
(v) In order to assess the degree of disorder-induced softening of the vibrational structure in glasses under investigation, place the values of the experimental NMD widths of all atomic species on a scale between their respective MBD values and the values obtained from the apVDOSs of the parent metal oxides. (The predictions of the DFT calculations for the values of the NMD widths of all types of atomic species present in the parent metal oxides reproduce the experimental NCS results very well, as evidenced by values listed in Tables S12–S16 in the SI.)

Oxygen is present in all parent metal oxides. In every oxide, the oxygen atoms are slightly differently bound. Thus, within the harmonic approximation, oxygen atoms in the lattices of different oxides have slightly different local binding potential shapes and, therefore, different values of the restoring force constants. Thus, for the oxygen, the upper limits of the values of the NMD widths were obtained from the linear combinations of the DFT predictions of all oxygen atoms in the parent metal oxide systems. The predictions of the DFT calculations for the values of the NMD widths of all types of atomic species present in the parent metal oxides reproduce the experimental NCS results very well (see Tables S12–S16 in the SI).

**Figure 8:**
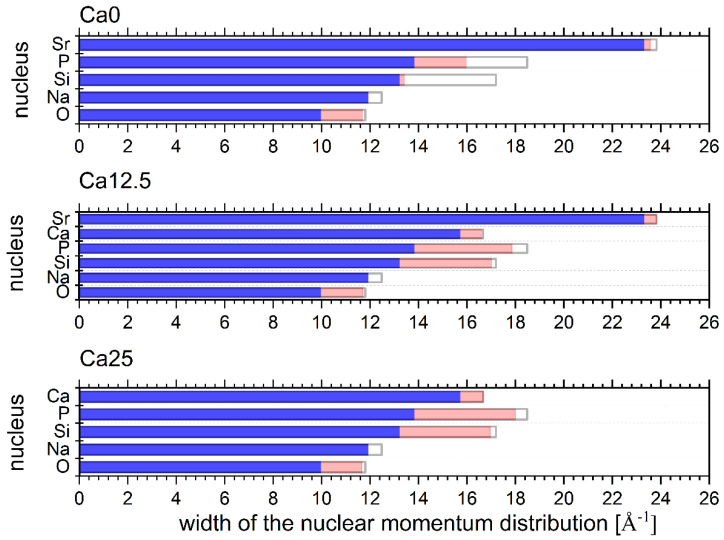
The disorder-induced softening of the widths of nuclear momentum distributions of individual atomic species present in bioactive glasses Ca0, Ca12.5, and Ca25. The bar charts show the adopted disorder scale: (i) the blue bars designate the Maxwell-Boltzmann distribution width limits for completely disordered gas of non-interacting particles without an underlying potential; (ii) the white bars show the upper distribution width limits calculated from atom-projected vibrational densities of states of parent metal oxides; and (iii) the red bars show the widths obtained from the analysis of the NCS experiments. See text for details.

As shown in Figure 8, by comparing the values of the NMD widths obtained from the experiments in glasses with their counterparts calculated from the DFT harmonic lattice dynamics simulation results in parent metal oxides, one can observe disorder-induced softening of all widths, albeit to a different extent in different glass samples. Before we proceed to the discussion of the disorder-induced NMD width softening in the Ca0, Ca12.5, and Ca25 glasses, it is worth noting that the values of the widths can be interpreted as Boltzmann population factor-weighted first moments of the atom-projected vibrational densities of states [18,36,37]. Thus, high-frequency vibrations contribute the most to the values of NMD widths at any given temperature. Any proportion of distorted (with respect to the local structure of parent metal oxides) bonds should decrease the frequencies of the associated vibrational modes. If those affected modes are the high-frequency stretching or bending ones, the effect of softening these modes on reducing the values of the NMD widths should be visible in NCS experiments [18].

The whole NCS data analysis, whose results are presented in Figure 7 and Figure 8, has been conducted based on the assumption that the masses of the recoiling species correspond to standard atomic weights of the elements. In reality, there is a distribution of isotopes for each atomic species. The isotopic mass disorder causes disorder of the bond lengths, and the disorder of the force constants propagating into the atom projected vibrational densities of states and NMD widths [18]. As far as the NCS data analysis is concerned, two effects are anticipated. The first one relates to the disorder-induced softening and broadening of the modes in the atom-projected vibrational densities of states. As the values of the NMD widths, according to Formula (5), are associated with the Boltzmann population factor-weighted centres of gravity of the atom-projected vibrational densities of states, it is more the mode softening than its broadening that will have an effect on the values of the NMD widths in the NCS spectra [18]. The mode softening will shift the centres of gravity to lower values compared to their counterparts in the parent metal oxides and thus cause the softening of the NMD values. Importantly, the analysis of Formula (4), which links the values of the NMD widths with the force-constant magnitudes, shows that, at room temperature, the values of the force constants are mostly sensitive to the variations of the isotopic mass and not to the variations of the NMD width values. Thus, any amount of uncertainty caused by the distribution of the isotopic masses of the same atomic species propagates into an associated uncertainty in the magnitude of the force constant, even if the NMD width values associated with different isotopes of the same atomic species are equal within the experimental accuracy. Taking into account only the isotopes whose abundance is big enough to be detected by the NCS technique, the silicon isotopes ^29^Si and ^30^Si, the calcium isotopes ^42^Ca, ^43^Ca, and ^44^Ca, and the strontium isotopes ^84^Sr, ^86^Sr, ^87^Sr, and ^88^Sr, should be taken into account as potential sources of the force constant and NMD width disorder and softening in the Ca0, Ca12.5, and Ca25 glasses. On the whole, mode softening and broadening, caused by the combined action of the positional, mass, and force-constant disorder, should be visible in the NCS spectra of the Ca0, Ca12.5, and Ca25 glasses when contrasted with their counterparts recorded for the parent metal oxides.

We will start our discussion with the analysis of data shown in Figure 8 by attempting to link the observed NMD widths to features observed in Raman data shown in Figure 6. In doing so, we must remember that in the absence of any ab initio calculation for the glasses, one cannot link the atom-projected vibrational densities of states in the glasses to the intensities of individual bands in the experimental Raman spectra. Certain Raman bands may be associated with displacements of selected atomic species. Conversely, displacements of many types of atomic species may contribute jointly to other Raman bands.

The Ca25 glass has a relatively weak Raman high-frequency band centred at 1367–1377 cm^−1^ due to P=O stretching vibrations. The Ca25 glass has the smallest value of the oxygen NMD width of 11.6 Å^−1^, compared to 11.7 Å^−1^ both in Ca12.5 and Ca0, where this band is of almost the same relative intensity but much stronger than in the case of the Ca25 glass. Thus, most likely, the P=O stretching vibrations have the highest contribution to the oxygen kinetic energy (and hence, the oxygen momentum distribution width) compared to the mid-frequency Si-O-Si, P-O-P, and Si-O-P bending vibration modes and Si-O and P-O stretching vibration modes.

Interestingly, the NMD width of P has its highest value for the Ca25 glass (18 Å^−1^), compared to 17.9 Å^−1^ in Ca12.5 and 16 in Ca0. This result can be understood assuming that the mid-frequency P-O-P and Si-O-P bending vibration modes and P-O stretching vibration modes have the highest contribution to the kinetic energy of phosphorus. These vibrations dominate in the case of the Ca25 and are still relatively more represented in the case of the Ca12.5 compared to the Ca0.

The Si NMD widths in the Ca25 and Ca12.5 glasses are 17 Å^−1^, whereas their counterpart in the Ca0 glass is 13.4 Å^−1^. Considering that all vibrational modes relevant to the Si motion are below 1110 cm^−1^, the substitution of CaO with SrO in the glass structure should not have much influence on the values of the kinetic energies of silicon atoms in all glass structures. Indeed, the Si NMD widths in the Ca25 and Ca12.5 glasses are very similar. However, a much lower value of the Si NMD width of the Ca0 glass compared to that in the Ca12.5 cannot be easily reconciled with similar distributions of Raman intensities in the Ca0 and Ca12.5. Thus, it may be caused by a numerical artefact due to the fitting of the NCS data containing overlapping recoil peaks of Na, Si, and Ca.

The Ca NMD widths are 16.7 and 16.6 Å^−1^ in the Ca25 and Ca12.5 glasses, respectively. The fact that the high-frequency band centred at 1367–1377 cm^−1^ is relatively weak in the sample containing 25 mol% CaO and increases dramatically in the Ca12.5 glass without affecting the value of the Ca NMD width signifies that the high-frequency vibrations do not contribute to the kinetic energy of calcium. Thus, the similarity of the Ca NMD widths in the Ca25 and Ca12.5 glasses should be caused by the similarity of their low-to-mid-frequency range vibration bands.

In the case of the Na, the values of the NMD widths are 11.9 Å^−1^ for all three glass systems under consideration. Thus, most likely, in the case of Na, the low-to-mid-frequency range of vibrations contributes most to the kinetic energy of Na.

Finally, in the case of strontium, the values of 23.8 and 23.6 Å^−1^ were obtained for the Ca12.5 and Ca0 glasses. The high-frequency Raman band of 1110–2000 cm^−1^ dramatically increases intensity upon replacing all the Ca^2+^ with Sr^2+^, but its shape remains relatively unaltered compared to the Ca25 glass (Figure 6). Hence, the vibrational modes in the 1110–2000 cm^−1^ region contribute the most to the kinetic energy of Sr. A small difference in the NMD width values between the Ca12.5 and Ca0 glasses could stem from a slightly more vibrational density of states in the low-to-mid-frequency region in the Ca12.5 glass.

The disorder-induced softening of NMD widths, as compared to their counterparts in parent metal oxides, has to be linked to the distributions of coordination numbers and bond lengths obtained from the RMC analysis of the total scattering data in Ca0, Ca12.5, and Ca25 glasses. Another physical interpretation of the NMD widths needs to be adopted to make this comparison more intuitive. Namely, instead of resorting to atom-projected vibrational densities of states, where every mode that contributes carries a certain amount of kinetic energy, a ‘mean-field’ picture can be used, whereby a mean effective local potential with its eigenstates and eigenvalues replaces a collection of quantum harmonic oscillators with different sets of eigenenergies. This effective local potential has a force constant associated with it and an effective frequency of vibration related to this force constant [19,20,38]. In a harmonic picture, the vibration frequency would be proportional to the square root of the ratio of the mean force constant and the effective mass of the vibrating species, and the value of the NMD width would be proportional to the square root of this frequency (the fourth root of the force constant). The effective force constant and the associated NMD width values can be related to the coordination number and bond lengths of atoms in the immediate neighbourhood of the atomic species vibrating in the effective potential.

The RMC analysis reveals that the Si-O covalent bond length is 1.60 ± 0.01 Å in the Ca25 and Ca12.5, while in the Ca0, the bond length is 1.65 ± 0.02 Å. The value obtained from the DFT-based geometry optimisation of the periodic SiO_2_ structure is 1.61 Å. Assuming that the effective force constant of the Si-O bond decreases with increasing bond length, the Si NMD width values for the Ca25 and Ca12.5 should be very similar to their counterparts in the SiO_2_ crystal, and indeed, the amount of the NMD width softening observed for the Ca25 and Ca12.5 is minimal. However, in the case of the Ca0 glass, the Si-O bond is much longer than in the SiO_2_ crystal, resulting in a much softer (smaller) value of the Si NMD width in the Ca0. Moreover, the average coordination number distribution shows that Si atoms are surrounded by four oxygen atoms, which is identical to the coordination number distribution in the SiO_2_ crystal. Thus, the spatial average of the Si-O bond length is similar in both types of systems, corroborating the trend in the Si NMD width values.

The P-O first neighbour distance is 1.60 ± 0.01 Å for all three glasses, and the P atoms are, on average, 4-fold coordinated by oxygen atoms. The P-O bond length distribution ranges from 1.44 to 1.58 Å in the P_2_O_5_ single crystal. Thus, there is a clear P NMD width softening in all three glasses compared to the P_2_O_5_ single crystal due to the much longer average P-O bond lengths in the glasses. However, in the case of the Ca0 glass, the degree of the softening is much larger than in the Ca12.5 and Ca25 glasses, which can result from a slight inaccuracy of the NCS data analysis in the Ca0 glass due to the recoil peak overlap.

The RMC analysis revealed Ca-O first neighbour distributions with a characteristic peak at 2.60 ± 0.02 Å with a shoulder peak at 2.25 Å only in the case of the Ca25 glass, while the coordination analysis showed, for all glasses, that more than five oxygen atoms surround the Ca atoms. The DFT-based geometry optimisation in the case of the CaO crystal yielded Ca-O bond lengths of 2.35 Å and 6-fold coordination of the Ca atom by oxygens. Based on the first neighbour distance analysis, it is thus difficult to reconcile a small degree of the Ca NMD width softening observed in the Ca25 and Ca12.5 glasses compared to the CaO crystal. Therefore, the competing effect of the bond length and coordination number distribution in both glasses has to affect the Ca NMD width.

The RMC analysis for the Sr-O distances shows an intensive peak at 2.60 ± 0.01 Å in the Ca0 and Ca12.5 glasses. The coordination number of Sr with oxygen atoms is 4.59 ± 0.05 in the Ca12.5 glass, increasing to 5.16 ± 0.05 for the Ca0 glass. In the case of the SrO single crystal, the DFT geometry optimisation yields the Sr-O bond length of 2.53 Å and 6-fold coordinated Sr atoms. The NCS data analysis reveals that the Sr NMD width softens in both glasses, but the degree of softening is much bigger in the Ca0 glass than in the Ca12.5 glass. In light of the RMC results, this effect seems to be caused by the increased Sr coordination with oxygen atoms in the Ca0 glass.

The Na-O distance analysis in the glasses using the RMC technique reveals a bimodal distribution peaked at characteristic distances of 2.20 ± 0.02 Å and 2.60 ± 0.02 Å. The DFT geometry optimisation of the Na_2_O crystal yields a Na-O bond of length of 2.35 Å. Interestingly, the NCS data analysis reveals that the degree of the Na NMD width softening in all three glasses is almost identical, with the width values being very close to the Maxwell–Boltzmann limit at room temperature. The softening of the NMD widths must be an effect of the overall increase in the Na-O distance in all three glasses compared to the Na_2_O crystal.

Finally, the RMC analysis reveals a distribution of the O-O distances characterised by two peaks at 2.60 ± 0.03 and 3.55 ± 0.05 Å and the O-O average coordination number close to 5 atoms. As the DFT-based calculation of the NMD width values for the oxygen in all three glasses is based on the averaged oxygen-projected vibrational densities of states in the parent metal-oxide single crystals, building an intuition based on a comparison between the local structure in the glasses and the metal oxides is difficult.

However, weighted over the glass sample stoichiometries, average values of the O-O distances in the parent metal oxides are 3.21, 3.18, and 3.15 Å in the Na0, Na12.5, and Na25 glasses. One can thus explain the softening of the NMD widths of the oxygen in all three glasses as an effect of systematically longer O-O average distances in the glasses as compared to all their parent metal oxides.

The values of the widths of nuclear momentum distributions, kinetic energies of individual atomic species, and effective force constants in the Ca0, Ca12.5, and Ca25 glasses are listed in Table 2. 

Amongst the effective mean forces acting locally on the atomic species in the CaO, Ca12.5, and Ca25 glasses, the highest magnitude is that of the local force acting on the Sr ions, followed by Ca, with the smallest values obtained for the oxygen atoms. This picture is consistent with the fact that no significant structural changes occur as Ca is replaced by Sr, with the main building blocks in the glass structures being the SiO_4_ and PO_4_ units. Namely, any additional local strains induced by the Sr for Ca replacement do not affect the structure. However, such changes in the local force distributions acting within the first coordination spheres of Ca and Sr are most likely responsible for the dynamical features appearing in the Raman spectra and the magnitudes of the NMD widths. The magnitude of the force-constant disorder of O, Na, and P are 0.01 eV/Å^2^ and 0.31, 0.07, and 0.63 eV/Å^2^ for Si, Sr, and Ca, respectively. This relatively big difference in the force-constant disorder magnitudes is caused by the fact that, within the harmonic approximation for the effective (mean-field) local potentials (Equation (2)), the force-constant magnitude at room temperature is much more sensitive to the isotopic mass distribution than to the distribution of NMD widths. The magnitudes of the force-constant disorder obtained here must be considered lower conservative bounds. Most likely, due to anharmonicity combined with isotopic mass disorder, the magnitude of the real force-constant disorder is greater.

## 3. Materials and Methods 

### 3.1. Sample Preparation

The raw materials used for the preparation of the bioglasses were all of purum p.a. grade: SiO_2_ (Umicore, 99.99% LOT:C000353436), CaO (Alfa Aesar, 99.95% LOT: S07D029), Na_2_O (Alfa Aesar, 99.9% LOT: W23C063), P_2_O_5_ (Alfa Aesar, 99.99% LOT: Z30B016) and SrO (Alfa Aesar, 99.5% LOT: N24D035).

The amorphous 45SiO_2_-(25-x)CaO-28Na_2_O-2P_2_O_5_-xSrO, x = 0; 12.5; 25 mol% samples were prepared by the melt-quenching technique. The samples were melted under atmospheric conditions between 1200 and 1250 °C in Pt crucibles. The glasses were obtained by pouring the melts onto a stainless-steel plate. The bulk samples were comminuted by ball-milling (Retsch MM400, Haan, Germany), using agate balls to a particle size below 50 μm. The synthesised and investigated samples are denoted as follows: Ca25 (for 45SiO_2_-25CaO-28Na_2_O-2P_2_O_5_), Ca12.5 (for 45SiO_2_-12.5CaO-28Na_2_O-2P_2_O_5_-12.5SrO), and Ca0 (for 45SiO_2_-28Na_2_O-2P_2_O_5_-25SrO).

### 3.2. Differential Thermal Analysis

The differential thermal analysis (DTA) was performed using a Setaram TG92-16.18 with a thermogravimetric analyser (TGA-92, Mettler-Toledo, Columbus, OH, USA) set up at controlled heating and cooling rates of 10 °C/min between room temperature and 1300 °C in an Al_2_O_3_ crucible under an Ar atmosphere. The DTA was calibrated by the melting of high-purity In, Zn, and Al metals. Glass transition temperature (T_g_), crystallisation temperature (T_c_) and melting temperature (T_m_) were determined from the DTA measurements.

### 3.3. Neutron Diffraction Measurements

Neutron diffraction (ND) experiments were performed on the PSD diffractometer (λ_0_ = 1.068 Å) [39] at the Budapest Neutron Centre in the momentum transfer range Q = 0.5–9.8 Å^−1^. PSD is a neutron powder diffractometer with a medium resolution of about 1% and high counting efficiency. It has been working at the refurbished 10 MW Budapest research reactor since 1994. The diffractometer uses a tangential thermal channel 9T with a diameter of 100 mm in the concrete shielding of the reactor. This geometry ensures a pure thermal neutron beam with low gamma and epithermal neutron flux. PSD uses a linear position-sensitive He detector system for data collection. The active length of the detector is 610 mm, and the diameter is 25 mm. The detector is filled up with 8 atm He gas, 4 atm Ar and 5% CO. The detector assembly is mounted on the diffractometer arm, and in the final configuration, it consists of 3 (max. 5) position-sensitive detectors. Three detectors are placed in the vertical plane, and two further detectors are placed behind them for experiments on small amounts of amorphous materials when high counting efficiency is needed and resolution requirements are not important. With a detector–sample distance of 1200 mm, the detector spans an angular range of 28 degrees. The complete diffraction spectrum is recorded up to the scattering angle of 110 degrees and is collected by moving the detector arm in four steps. In the particular case of the neutron diffraction experiments on strontium-substituted bioactive glasses, the powder specimens of about 3–3.5 g were filled in thin-walled cylindrical vanadium sample holders of 8 mm diameter. Data were corrected for detector efficiency, background scattering, and absorption effects. The total structure factors, *S*(*Q*), were evaluated from the raw experimental data using the program package available at the facility.

### 3.4. Reverse Monte Carlo Simulations

The Reverse Monte Carlo (RMC) simulation is a powerful technique for building 3D structural models in accordance with experimental data. Total structure factors, S(Q), obtained from diffraction experiments, were simulated by the RMC^++^ program [40]. The RMC algorithm used in the RMC^++^ calculates the one-dimensional partial atomic pair correlation functions g_ij_(r) that are inverse Fourier transformed to calculate the partial structure factors, S_ij_(Q):

(1) $$S_{ij}=1+\frac{4\pi \rho_{0}}{Q}\int_{0}^{r_{max}}r\left[g_{ij}\left(r\right)-1\right]\sin\left(Qr\right)dr$$

where r_max_ is the half edge-length of the simulation box. The atomic configuration is modified by moving the atoms randomly until the calculated S(Q) agrees with the experimental data within the experimental error. The initial configuration was generated by a random distribution of 10,000 atoms in a cubic simulation box. The experimentally measured number density ρ_0_ was 0.0719, 0.0714, and 0.0680 atoms·Å^−3^ (corresponding to box edges of 25.90 Å, 25.96 Å, and 26.39 Å) for the Ca25, Ca12.5, and CaO samples, respectively. In the RMC simulation, constraints were used for the minimum interatomic distances between atom pairs (cutoff distances) to avoid unreasonable atom contacts and connectivity constraints. The starting cutoff distances were taken from previous studies [23,24,31,41,42]. During each run, the cutoff distances were modified, assuring realistic pair-distribution functions of interatomic distances, g_ij_(r). Close to 25 atomic configurations were obtained from the RMC calculations of each sample, which corresponded to more than 1,100,000 accepted atomic movements inside the simulation box.

### 3.5. Neutron Compton Scattering (NCS)

NCS experiments were performed at the VESUVIO spectrometer at the ISIS neutron and muon spallation source at the UK Rutherford Appleton Laboratory in Oxfordshire [20,43,44,45,46]. The measurements of all three glass samples were carried out at room temperature. The powder samples were placed into thin-walled flat aluminium cells. The cells were assembled out of two flat (one front and one backside) walls, each of a cross-section of 40 square cm, separated by an aluminium spacer of 3 mm thickness, and fully exposed to the incident VESUVIO neutron beam when placed perpendicular to its direction. The general setup of VESUVIO was described elsewhere [20,43,44,45,46]. VESUVIO is an inverted geometry instrument. The incident neutron beam has the intensity of 10^7^ neutrons/cm^2^/s at the ISIS power of 160 microAmperes of the proton beam to the neutron target. The incident neutron beam produced by the target enters a water moderator kept at the temperature of 300 K. It exits the water moderator as a white beam that contains neutrons of energies between 1 meV and 1000 eV and faces the neutron monitor that measures the incident neutron flux ca. 2.5 m in front of the sample position. The incident beam distance from the exit of the water moderator to the sample position is 11 m. The incident beam shape at the sample position is circular with a diameter of ca. 5 cm. When the deep inelastic scattering detection mode is selected on VESUVIO, the broadband incident neutron beam scatters off the sample, and its final neutron energy is fixed using resonant neutron absorption in gold foils at the energy of ca. 4.9 electron-Volts. The neutron detectors are placed in front (forward scattering) and rear (backscattering) sample positions, with distances between the sample positions and detectors varying from 0.5 to 0.7 m. The NCS spectra are recorded by detectors placed at different scattering angles in the form of histograms with the count-rate as a function of the time-of-flight (TOF) of the neutrons from their origin at the exit of the water moderator to the detector faces. The neutrons transmitted through the sample are measured by the transmission neutron monitor at ca. 2.4 m behind the sample position, and then the neutrons reach a beam stop. In the particular case of the NCS experiments on strontium-substituted bioactive glasses, the mass-resolved NCS spectra were recorded in the neutron time-of-flight (TOF) domain by detectors placed at scattering angles between 130 and 170 degrees (referred to as the backscattering regime). The NCS spectra were assumed to consist of recoil peaks with underlying purely Gaussian nuclear momentum distributions (NMDs) with standard deviations σ_M_ (where M is the mass of a given nucleus), hereinafter referred to as the NMD widths.

Each NCS spectrum C(θ,t) was recorded in the time-of-flight domain t as a function of a scattering angle θ. The NCS spectra were assumed to consist of recoil peaks with underlying purely Gaussian nuclear momentum distributions (NMDs), $J_{M}\left(x_{M}\left(t\right)\right),\;$ with standard deviations, σ_M_ (where M is the mass of a given nucleus), hereinafter referred to as the NMD widths. The derivation of the theoretical expressions linking the C(θ,t) and $J_{M}\left(x_{M}\left(t\right)\right)$ is widely discussed in the literature [16]. Thus, only a brief summary will be given here. Within the so-called Impulse Approximation (IA), the NCS spectrum in the TOF domain is given by [16]:

(2) $$C\left(\theta ,t\right)=\frac{E_{0}\left(t\right)I\left(\left(t\right)\right)}{q\left(\theta ,t\right)}\sum_{M}Mc_{M}J_{M}\left(x_{M}\left(\theta ,t\right)\right)⨂R_{M}\left(x_{M}\left(\theta ,t\right)\right)$$

where E_0_ and I(E_0_) are the incident neutron energy and incident neutron spectrum, respectively, and q is the neutron momentum transfer. Furthermore, the effect of the M-dependent resolution function of the instrument is incorporated as a convolution, $J_{M}\left(x_{M}\left(\theta ,t\right)\right)⨂R_{M}\left(x_{M}\left(\theta ,t\right)\right)$.

The variable xM(θ,t)=yM(θ,t)σM2 is a scaled and centred magnitude of the longitudinal momentum $y_{M}\left(\theta ,t\right)\;$ of a nucleus of mass M, expressed in units of inverse Angstroms. The integrated intensity of each recoil peak is proportional to the product of the total bound scattering cross-section, $A=4\pi {b_{M}}^{2}$ (where $b_{M}$ is the total bound scattering length of a nucleus) and the number of atoms with mass M per sample formula unit, $N_{M}$. The NMD of each mass present in the NCS spectrum was  corrected for the departure from the IA using a third-order Hermite polynomial, $H_{3}\left(x_{M}\right)$ and expressed as [16]:

(3) $$J_{M}\left(x_{M}\left(\theta ,t\right)\right)=\frac{1}{\sqrt{2\pi \sigma_{M}^{2}}}e^{-x_{M}^{2}\left(\theta ,t\right)}\left(1-\frac{\sqrt{2}}{12}\frac{\sigma_{M}}{q\left(\theta ,t\right)}H_{3}\left(x_{M}\left(\theta ,t\right)\right)\right)$$

Crucially for the assessment of the force-constant disorder, the values of σ_M_ can be used to calculate the magnitudes of the effective force constants, k_M_, acting on nuclei in silicate glasses. In order to obtain the values of k_M_, one can employ the theory of the mean force (MF) function, with the MF defined as the average force acting on an atomic particle by keeping all other particles in the system fixed [19]. In the case of an isotropic harmonic potential, the MF is linear with atomic displacement. In such a case, the force-constant magnitude for a nucleus of mass M at temperature T can be calculated using the formula [19]:

(4) $$k_{M}=\frac{{\left(k_{B}T\right)}^{2}M}{\hbar^{2}}-k_{B}T\sigma_{M}^{2}$$

where k_B_ is the Boltzmann constant. Importantly, Formula (4) is not valid in the limit of T = 0 due to the limitations of the sequence of Feynman–Trotter approximations to the thermal Feynman path integral for a general non-relativistic system characterised by a smooth, single-minimum interaction potential that underlies a momentum distribution of a given nucleus [19]. However, as Formula (4) has been successfully applied to the description of the mean force in polycrystalline and amorphous ice at 100 K [19], it will be used here to describe the mean forces of glasses and their parent metal oxides at 300 K [18]. As mentioned in our previous work [18], in an NCS experiment, each recoil peak needs to be fitted with an underlying momentum distribution defined for a single atomic species with a standard atomic weight, and the entire effect of the isotopic mass disorder is contained in the uncertainty of the fitted NMD width value, which is broadened due to the combined effect of the isotopic mass distribution and the nuclear momentum distribution of each isotope of the same atomic species present in a sample. Thus, in what follows, we will account for the NMD width disorder in the glass samples under investigation by simply using the mean values of the NMD widths together with the experimental uncertainties, as obtained directly from NCS experiments. The uncertainties of NMD widths will be assumed to stem from normal distributions of NMD width values, in agreement with standard NCS data analysis schemes [20,46,47,48]. The mean values and the standard deviations of the force-constant distributions will then be calculated using the values of their counterparts obtained for the NMD widths by error propagation.

### 3.6. Raman Spectroscopy

A Renishaw In-Via Reflex micro-Raman spectrometer (Wotton-under-Edge, UK) was used to measure the Raman spectra of the glasses by using a diode laser of 785 nm excitation wavelength, an edge filter for Stokes spectra, and a Peltier cooled CCD detector. Measurements were performed in an unpolarised mode at room temperature in the backscattering geometry in the Raman shift range of 300 to 2200 cm^−1^ at a spectral resolution of ~1 cm^−1^.

### 3.7. Density Functional Theory

For the constituent metal-oxide single crystals (CaO, Na_2_O, P_2_O_5_, SiO_2_, and SrO), electronic structure calculations under Periodic Boundary Conditions (PBCs) and employing the Plane-Wave Pseudo-Potential (PW-PP) formulation of Density Functional Theory (DFT) were performed in the CASTEP 21 package [49,50] used for the input and output preparation the Materials Studio2021^®^ software. The initial structure of P_2_O_5_ was downloaded from the ICSD database [ICSD Entry: 16,611, [51]], and the initial structures of all other metal oxides were imported from the Materials Studio internal library. After the geometry optimisation, the vibrational responses [the phonon dispersion, as well as the total (VDoSs) and partial (atom-projected) vibrational density of states (apVDoSs) for each constituent atomic species type] were calculated within the Harmonic Lattice Dynamics (HLD) approach. Exchange correlation was treated within the Local Density Approximation (LDA) with the Koelling–Harmon treatment of relativistic effects and sets of on-the-fly-generated norm-conserving PPs describing the core electrons. The electronic wave functions were computed using a PW basis set with a kinetic energy cutoff of 1000 eV. The Monkhorst–Pack grid with the k-point spacing of 0.07 Å^−1^ was sufficient for well-converged phonon properties.

Geometry optimisation calculations of the unit cells of the constituent metal-oxide crystals were performed under atmospheric pressure, using the LBFGS algorithm and imposing a three-point finite basis set correction. The convergence criteria in the self-consistent field (SCF) variation were set to 5.0 × 10^−7^ eV/atom. During the geometry optimisation, total energy convergence tolerance was set to 5.0 × 10^−7^ eV/atom, maximum ionic force tolerance to 10^−2^ eV/Å, maximum ionic displacement tolerance to 5.0 × 10^−4^ Å, and maximum stress component tolerance to 2.0 × 10^−2^ GPa.

The formulation of the Density Functional Perturbation Theory (DFPT) in the reciprocal space was employed in CASTEP 21 in the limit of T = 0 K to calculate phonon-dispersion relations across the first Brillouin Zone (BZ), VDoSs, and apVDoSs. Phonon convergence tolerance of 10^−5^ eV/Å^2^ was used.

The widths (standard deviations) of the nuclear momentum distributions of the individual atomic species were calculated based on the apVDoSs following the formalism already established in our previous work [18,20,36,37]. To this end, the first moments of the apVDoSs $g_{M}\left(E\right)$, weighted by the Botzmann population factors coth(E2kBT), were calculated to predict the values of the kinetic energies $E_{kin,M}$ and the widths of the nuclear momentum distributions $\sigma_{M}$ at room temperature, according to the following formula:

(5) Ekin,M=34∫0∞EgM(E)coth(E2kBT)dE

where Ekin,M=3ℏ2σM22M, *k* is the Boltzmann constant, and E is the vibrational energy.

For the calculation of the kinetic energy and NMD width values, the NCS-specific system of units was used [18,20,36,37]. The energy and energy transfer values were expressed in units of meV, the nuclear momenta in Å^−1^, and nuclear masses in atomic mass units (amu), with the Planck constant, ħ, value of 2.044 amu^½^ meV^½^ Å.

The results of the DFT calculations for all parent metal oxides, including the geometry optimisation and phonon calculation results, are summarised in the Supplementary Information (SI). Moreover, the SI shows the comparison of the experimental values of the NMD widths of all constituent atomic species with their DFT-computed counterparts.

## 4. Conclusions

The main goal of this work was to investigate the influence of SrO substitution in the local structure and dynamics in Si-Ca-Na-P oxide glasses. The SrO (x = 0, 12.5, 25) was substituted to CaO (25-x) in a molar percentage. SrO-containing glasses showed lower glass transition, melting, and crystallisation temperatures. The addition of the Sr^2+^ ions decreased the thermal stability of the glass structure. The total neutron diffraction augmented by the RMC simulation revealed that Sr played a similar role as Ca in the glass structure when substituted on a molar basis. The bond length and the coordination number distributions of the network modifiers and network formers did not change when SrO (x = 0, 12.5, 25) was substituted for CaO (25-x). However, the network connectivity increased in Ca12.5 glass due to the increase in the Si-O-Si interconnected chain. The changes in the local structure introduced by the SrO substitution were clearly visible in Raman spectra in all glasses, and the values of the NMD widths of all atomic species present in the glasses could be reconciled with the features present in the Raman spectra. Moreover, the values of the NMD widths were observed to soften with respect to their counterparts, which were calculated using the DFT-based harmonic lattice dynamics for parent metal oxides. This softening was attributed to the combined effect of the positional, isotopic, and force-constant disorder in the glasses. The amount of the force-constant disorder was assessed based on the distributions of the NMD widths obtained from the analysis of the neutron Compton spectra in glasses. On the whole, the combination of the structural and dynamic probes employed in this study has proven to be very effective in describing the local structure and dynamics of bioactive glasses. The presented work also has a practical dimension. It addresses the question of how the improvement in the material properties that are directly relevant to its biological function (such as the osteoblast activity, inhibiting osteoclast function, as well as the anti-inflammatory and bone growth enhancement) correlates with the changes in mechanical and thermodynamic properties of these materials. Investigating this correlation will help find the optimal degree of strontium substitution in bioactive glasses that will provide the best possible enhancement of their biological function without affecting their mechanical and thermodynamic properties.

## Figures and Tables

**Table 1:** The characteristic temperatures of the bioactive glass compositions.

Glass ID	T_g_ (°C)	T_c_ (°C)	T_m_ (°C)	T_onset c_-T_g_ (°C)	T_onset M_ (°C)	ΔH_M_ (J/g)
Ca25	508	685	1189	104	1086	238
Ca12.5	473	710	1120	129	1031	212
Ca0	455	621	1112	134	1020	146

**Table 2:** The values of the experimental widths of nuclear momentum distributions, kinetic energies of individual atomic species, and force constants in the Ca0, Ca12.5, and Ca25 glasses. See text for details.

σ (Å^−1^) E_kin_ (meV) k (eV/Å^2^)			
Nucleus	Ca0	Ca12.5	Ca25
O	11.69 ± 0.24 53.6 ± 2.2 2.56 ± 0.01	11.71 ± 0.21 53.7 ± 1.9 2.56 ± 0.01	11.66 ± 0.31 53.3 ± 2.8 2.56 ± 0.01
Na	11.92 ± 0.41 38.7 ± 2.7 3.67 ± 0.01	11.92 ± 0.41 38.7 ± 2.7 3.67 ± 0.01	11.92 ± 0.41 38.7 ± 2.7 3.67 ± 0.01
Si	13.42 ± 0.79 40.3 ± 4.8 4.48 ± 0.31	17.02 ± 0.53 64.9 ± 4.0 4.48 ± 0.32	16.96 ± 0.66 64.41 ± 5.0 4.48 ± 0.32
P	15.97 ± 1.13 51.6 ± 7.3 4.96 ± 0.01	17.86 ± 1.13 64.5 ± 8.2 4.96 ± 0.01	18.00 ± 0.89 65.5 ± 6.5 4.96 ± 0.01
Sr	23.56 ± 0.26 39.7 ± 0.9 14.00 ± 0.07	23.82 ± 0.40 40.60 ± 1.36 14.00 ± 0.07	-----
Ca	-----	16.62 ± 0.16 43.3 ± 0.8 6.40 ± 0.63	16.66 ± 0.40 43.5 ± 2.1 6.39 ± 0.63

## Data Availability

The data presented in this study are available upon reasonable request from the corresponding author. The raw neutron data collected at ISIS Neutron and Muon Source are related to the RB number: 2220011.

## Supplementary Materials

The following are available online at https://www.mdpi.com/article/10.3390/molecules29194720/s1, Table S1. Results of the DFT-based geometry optimisation for CaO. Table S2. Fractional coordinates in the DFT-optimised unit cell of CaO. Table S3. Results of the DFT-based geometry optimisation for Na_2_O. Table S4. Fractional coordinates in the DFT-optimised unit cell of Na_2_O. Table S5. Results of the DFT-based geometry optimisation for P_2_O_5_. Table S6. Fractional coordinates in the DFT-optimised unit cell of P_2_O_5_. Table S7. Results of the DFT-based geometry optimisation for SiO_2_. Table S8. Fractional coordinates in the DFT-optimised unit cell of SiO_2_. Table S9. Results of the DFT-based geometry optimisation for SrO. Table S10. Fractional coordinates in the DFT-optimised unit cell of SrO. Figure S1. Electronic band structure from the DFT calculation for CaO. Figure S2. Electronic band structure from the DFT calculation for Na_2_O. Figure S3. Electronic band structure from the DFT calculation for P_2_O_5_. Figure S4. Electronic band structure from the DFT calculation for SiO_2_. Figure S5. Electronic band structure from the DFT calculation for SrO. Figure S6. Total VDOS from the DFT calculation for CaO. Figure S7. Partial Ca-projected VDOS from the DFT calculation for CaO. Figure S8. Partial O-projected VDOS from the DFT calculation for CaO. Figure S9. Total VDOS from the DFT calculation for Na_2_O. Figure S10. Partial Na-projected VDOS from the DFT calculation for Na_2_O. Figure S11. Partial O-projected VDOS from the DFT calculation for Na_2_O. Figure S12. Total VDOS from the DFT calculation for P_2_O_5_. Figure S13. Partial P-projected VDOS from the DFT calculation for P_2_O_5_. Figure S14. Partial O-projected VDOS from the DFT calculation for P_2_O_5_. Figure S15. Total VDOS from the DFT calculation for SiO_2_. Figure S16. Partial Si-projected VDOS from the DFT calculation for SiO_2_. Figure S17. Partial O-projected VDOS from the DFT calculation for SiO_2_. Figure S18. Total VDOS from the DFT calculation for SrO. Figure S19. Partial Sr-projected VDOS from the DFT calculation for SrO. Figure S20. Partial O-projected VDOS from the DFT calculation for SrO. Figure S21. The NCS data of CaO. Table S11. Comparison of experimental and simulated values of the NMD widths for CaO. Figure S22. The NCS data of Na_2_O. Table S12. Comparison of experimental and simulated values of the NMD widths for Na_2_O. Figure S23. The NCS data of SrO. Table S13. Comparison of experimental and simulated values of the NMD widths for SrO. Figure S24. The NCS data of SiO_2_. Table S14. Comparison of experimental and simulated values of the NMD widths for SiO_2_. Figure S25. The NCS data of P_2_O_5_. Table S15. Comparison of experimental and simulated values of the NMD widths for P_2_O_5_.
