# Supplementary material for: Structural and Dynamical Effects of the CaO/SrO Substitution in Bioactive Glasses
Source: Molecules. 2024 Oct 5;29(19):4720. doi: 10.3390/molecules29194720 (PMC11478278; doi:10.3390/molecules29194720)
Supplement: Supplementary file 1 [file molecules-29-04720-s001.zip › molecules-3179800-supplementary.pdf]

Structural and Dynamical Effects of the CaO/SrO Substitution in Bioactive Glasses

Supplementary Information

Margit Fabian [1], Matthew Krzystyniak [2,*], Atul Khanna [3] and Zsolt Kovacs [4]

[1]HUN-REN Centre for Energy Research, Konkoly Thege Miklos st. 29-33, 1121 Budapest, Hungary

[2]ISIS Facility, Rutherford Appleton Laboratory, Chilton, Didcot OX11 0QX, UK

[3]Department of Physics, Guru Nanak Dev University, Amritsar 143005, India

[4]Department of Materials Physics, Institute of Physics, Eötvös Loránd University, Pázmány Péter st. 1/a, H-1117 Budapest, Hungary

* Correspondence: matthew.krzystyniak@stfc.ac.uk

1. Density Functional Theory geometry optimisation results for parent metal oxides

1.1 CaO

**Lattice parameters (Å)**	**Cell Angles**	**Cell Volume ($Å^3$)**	**Density**
a = 4.707598	alpha = 90.0	104.327335	2.150117 amu/ $Å^3$== 3.570354 g/cm^3
b = 4.707598	beta = 90.0		
c = 4.707598	gamma = 90.0		

Table S1. Results of the DFT-based geometry optimisation for CaO.

		Fractional coordinates		
element	Atom number	u	v	w
O	1	0.5	0.5	0.5
O	2	0.5	1.0	1.0
O	3	1.0	0.5	1.0
O	4	1.0	1.0	0.5
Ca	1	0.0	0.0	0.0
Ca	2	0.0	0.5	0.5
Ca	3	0.5	0.0	0.5
Ca	4	0.5	0.5	0.0

Table S2. Fractional coordinates in the DFT-optimised unit cell of CaO.

1.2 Na_2O

**Lattice parameters (Å)**	**Cell Angles**	**Cell volume ($Å^3$)**	**density**
a = 5.429477	alpha = 90.0	160.056750	1.548926 amu/ $Å^3$ = 2.572051 g/cm^3
b = 5.429477	beta = 90.0		
c = 5.429477	gamma = 90.0		

Table S3. Results of the DFT-based geometry optimisation for Na_2O.

		Fractional coordinates		
element	Atom number	u	v	w
O	1	0.0	0.0	0.0
O	2	0.0	0.5	0.5
O	3	0.5	0.0	0.5
O	4	0.5	0.5	0.0
Na	1	0.25	0.25	0.25
Na	2	-0.25	-0.25	0.25
Na	3	-0.25	0.25	-0.25
Na	4	0.25	-0.25	-0.25
Na	5	0.25	0.25	-0.25
Na	6	-0.25	-0.25	-0.25
Na	7	-0.25	0.25	0.25
Na	8	0.25	-0.25	0.25

Table S4. Fractional coordinates in the DFT-optimised unit cell of Na_2O.

1.3 P_2O_5

**Lattice parameters (Å)**	**Cell Angles**	**Cell volume (Å^3)**	**density**
a = 9.051766	alpha = 90.0	313.665687	1.810118 amu/ Å^3 = 3.005772 g/cm^3
b = 7.108968	beta = 90.0		
c = 4.874467	gamma = 90.0		

Table S5. Results of the DFT-based geometry optimisation for P_2O_5.

		Fractional coordinates		
element	Atom number	u	v	w
O	1	0.130789	0.28216	-0.0017
O	2	0.369211	0.78216	0.001696
O	3	-0.13079	-0.28216	0.498304
O	4	0.630789	0.21784	0.501696
O	5	-0.13079	-0.28216	0.001696
O	6	0.630789	0.21784	-0.0017
O	7	0.130789	0.28216	0.501696
O	8	0.369211	0.78216	0.498304
O	9	0.275028	0.504909	0.25
O	10	0.360545	0.154019	0.25
O	11	0.557792	0.581296	0.25
O	12	0.224972	1.004909	-0.25
O	13	0.139455	0.654019	-0.25
O	14	-0.05779	1.081296	-0.25
O	15	-0.27503	-0.50491	0.75

O	16	-0.36055	-0.15402	0.75
O	17	-0.55779	-0.5813	0.75
O	18	0.775028	-0.00491	0.25
O	19	0.860545	0.345981	0.25
O	20	1.057792	-0.0813	0.25
P	1	0.240015	0.287144	0.25
P	2	0.405917	0.649456	0.25
P	3	0.259985	0.787144	-0.25
P	4	0.094083	1.149456	-0.25
P	5	-0.24002	-0.28714	0.75
P	6	-0.40592	-0.64946	0.75
P	7	0.740015	0.212856	0.25
P	8	0.905917	-0.14946	0.25

Table S6. Fractional coordinates in the DFT-optimised unit cell of P_2O_5.

1.4. SiO_2

**Lattice parameters (Å)**	**Cell Angles**	**Cell volume (Å^3)**	**density**
a = 4.913000	alpha = 90.0	112.988938	1.595315amu/ Å^3 = 2.649082g/cm^3
b = 4.913000	beta = 90.0		
c = 5.405200	gamma = 120.0		

Table S7. Results of the DFT-based geometry optimisation for SiO_2.

		Fractional coordinates		
element	Atom number	u	v	w
O	1	0.411956	0.272165	0.218578
O	2	-0.27217	0.13979	0.551911
O	3	-0.13979	-0.41196	0.885244
O	4	0.272165	0.411956	-0.21858
O	5	0.13979	-0.27217	0.448089
O	6	-0.41196	-0.13979	0.114756
Si	1	0.467476	0	0.333333
Si	2	0	0.467476	0.666667
Si	3	-0.46748	-0.46748	1

Table S8. Fractional coordinates in the DFT-optimised unit cell of SiO_2.

1.4. SrO

**Lattice parameters (Å)**	**Cell Angles**	**Cell volume (A^3)**	**density**
a = 5.052830	alpha = 90.0	129.004232	3.212887 amu/ $Å^3$ = 5.335124 g/cm^3
b = 5.052830	beta = 90.0		
c = 5.052830	gamma = 90.0		

Table S9. Results of the DFT-based geometry optimisation for SrO.

		Fractional coordinates		
element	Atom number	u	v	w
O	1	0.5	0.5	0.5
O	2	0.5	1	1
O	3	1	0.5	1
O	4	1	1	0.5
Sr	1	0	0	0
Sr	2	0	0.5	0.5
Sr	3	0.5	0	0.5
Sr	4	0.5	0.5	0

Table S10. Fractional coordinates in the DFT-optimised unit cell of SrO.

2. Density Functional Theory band structure results for parent metal oxides

2.1 CaO

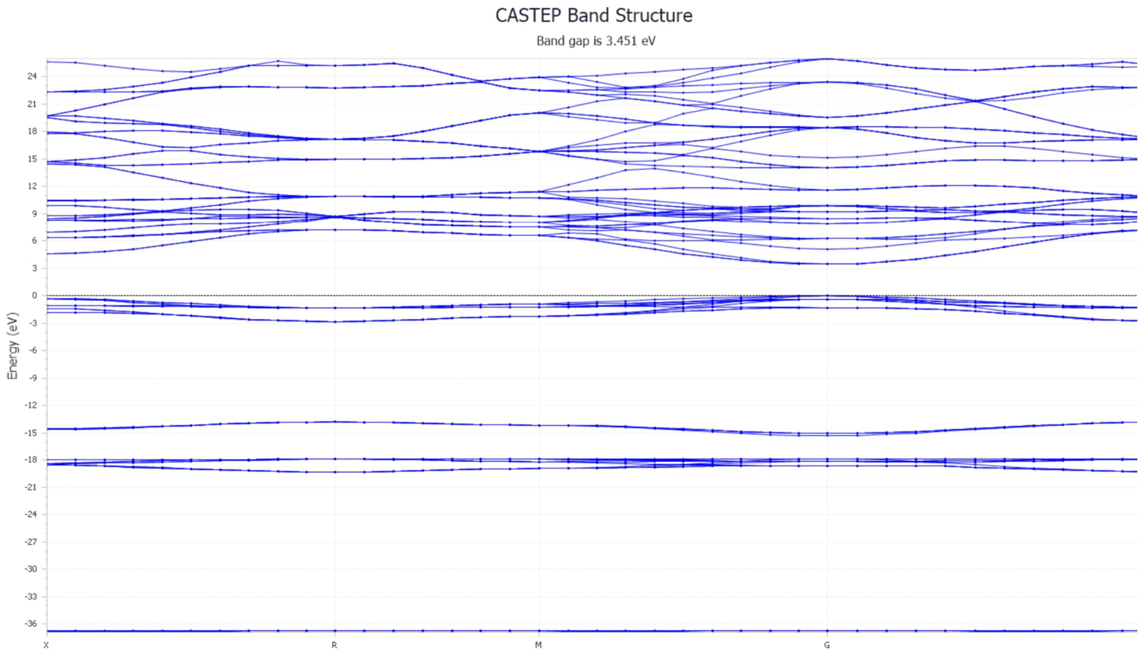

Figure S1. Electronic band structure from the DFT calculation for CaO.

2.2 Na_2O

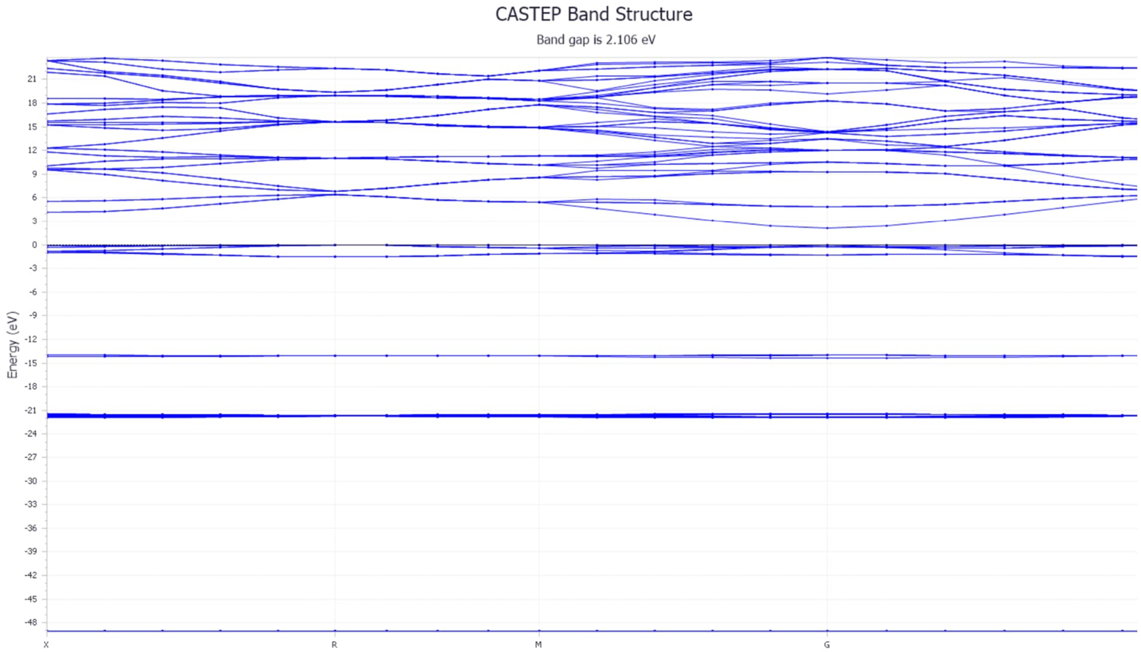

Figure S2. Electronic band structure from the DFT calculation for Na_2O.

2.3 P_2O_5

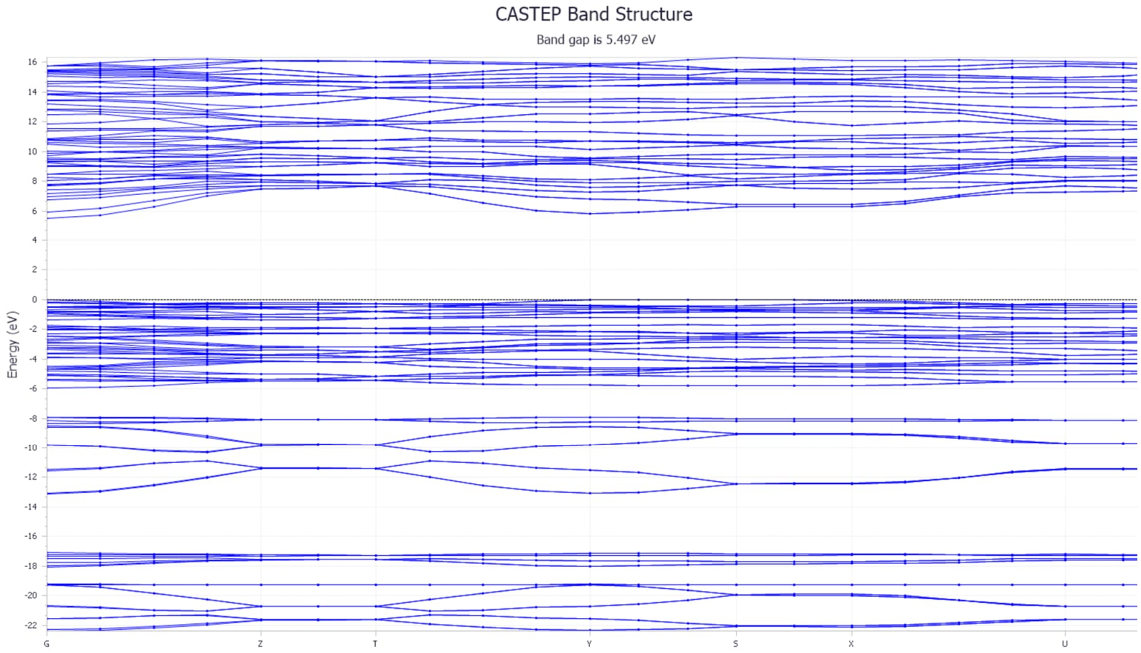

Figure S3. Electronic band structure from the DFT calculation for P_2O_5.

2.4 SiO_2

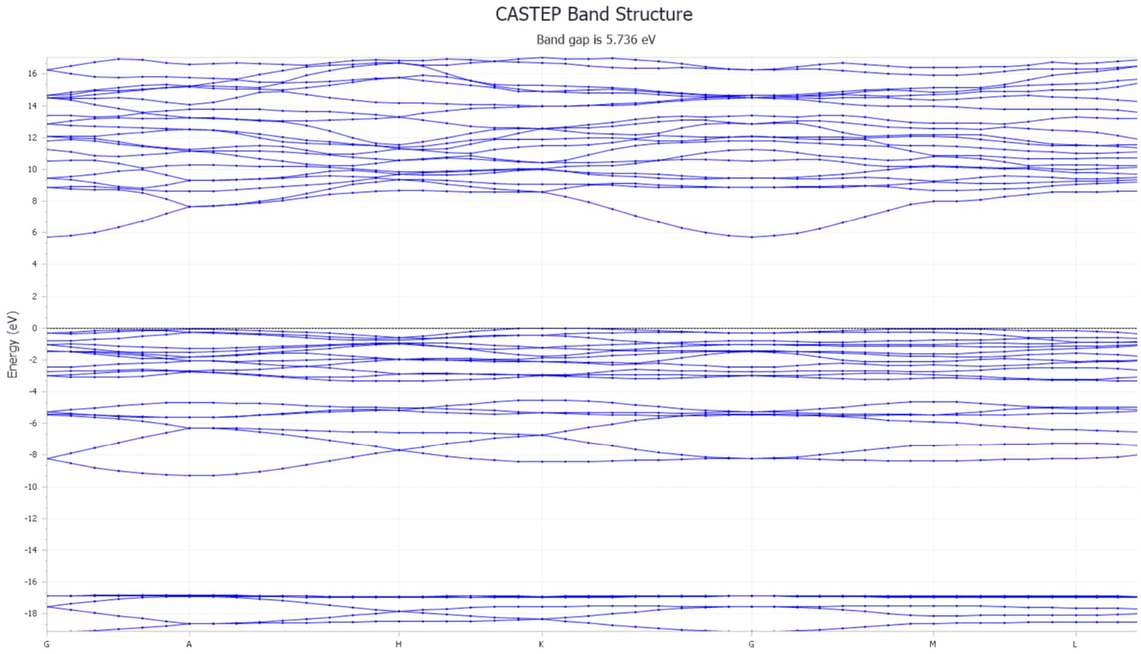

Figure S4. Electronic band structure from the DFT calculation for SiO_2.

2.5 SrO

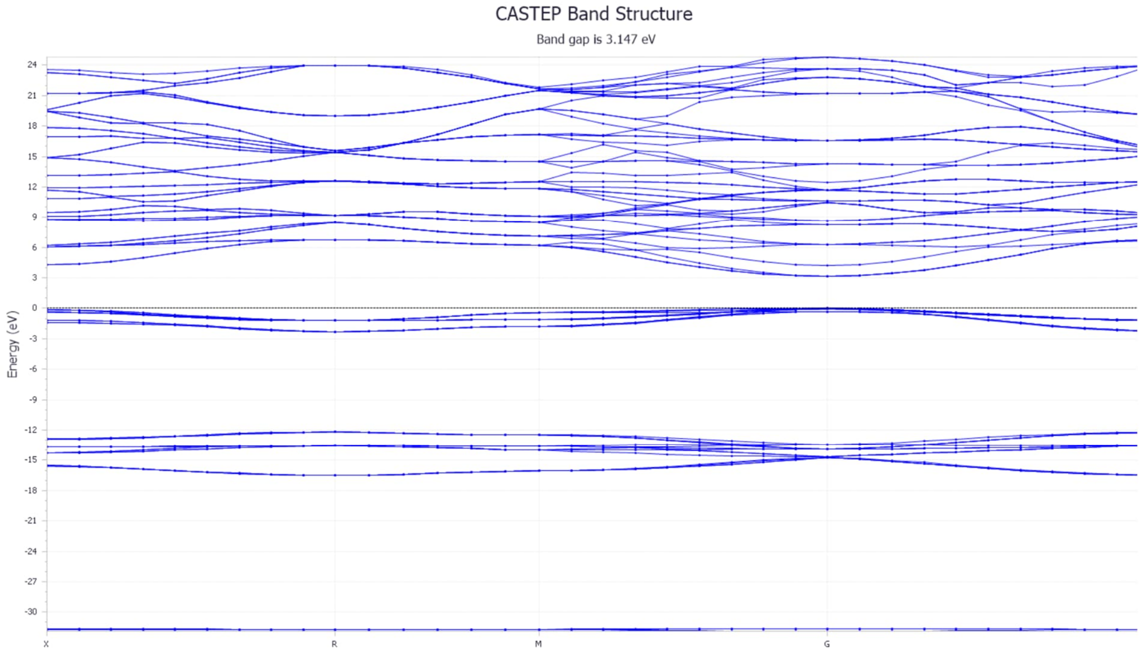

Figure S5. Electronic band structure from the DFT calculation for SrO.

3. Density Functional Theory phonon calculation results for parent metal oxides

3.1 CaO

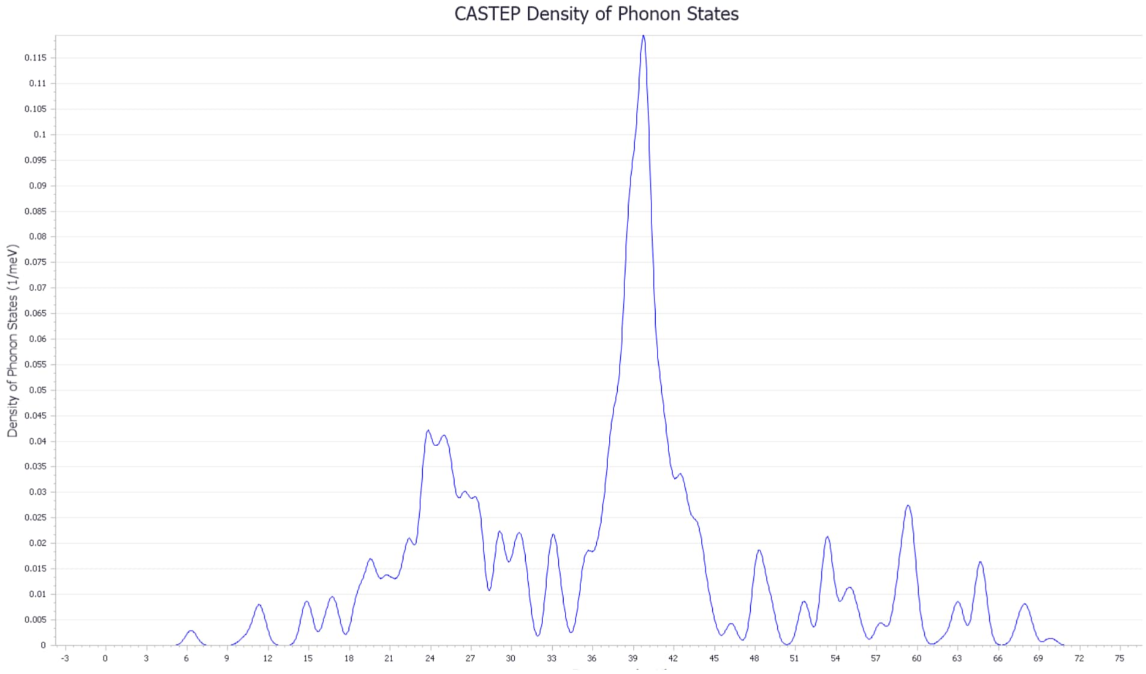

Figure S6. Total VDOS from the DFT calculation for CaO.

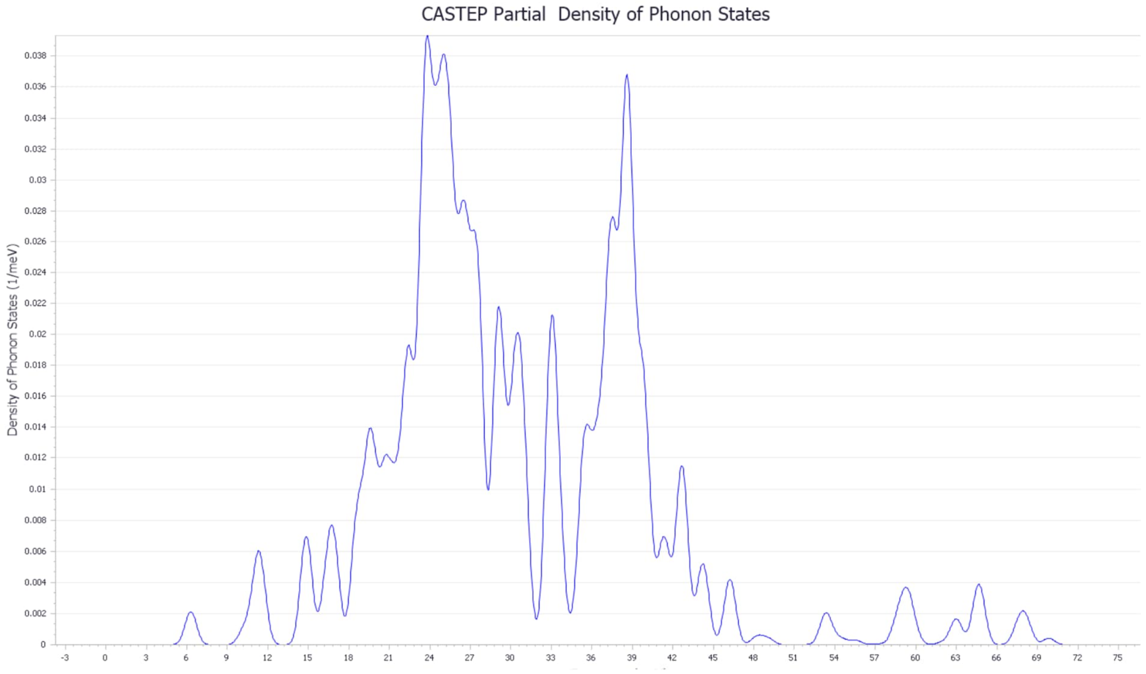

Figure S7. Partial Ca-projected VDOS from the DFT calculation for CaO.

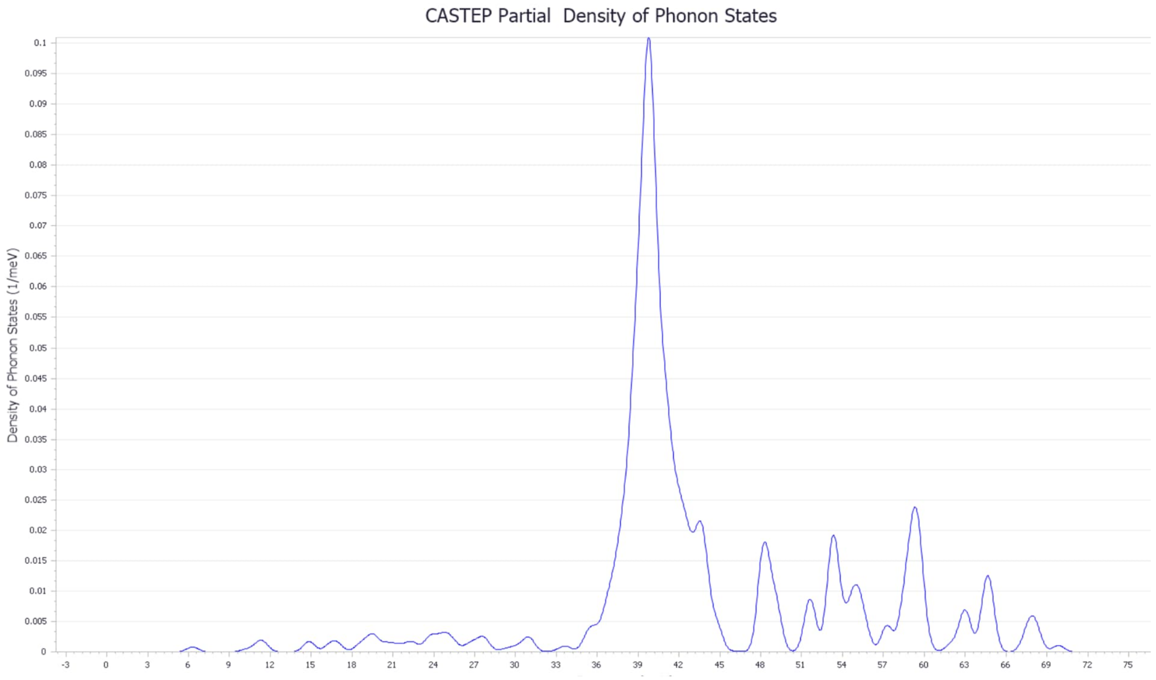

Figure S8. Partial O-projected VDOS from the DFT calculation for CaO.

3.2 Na_2O

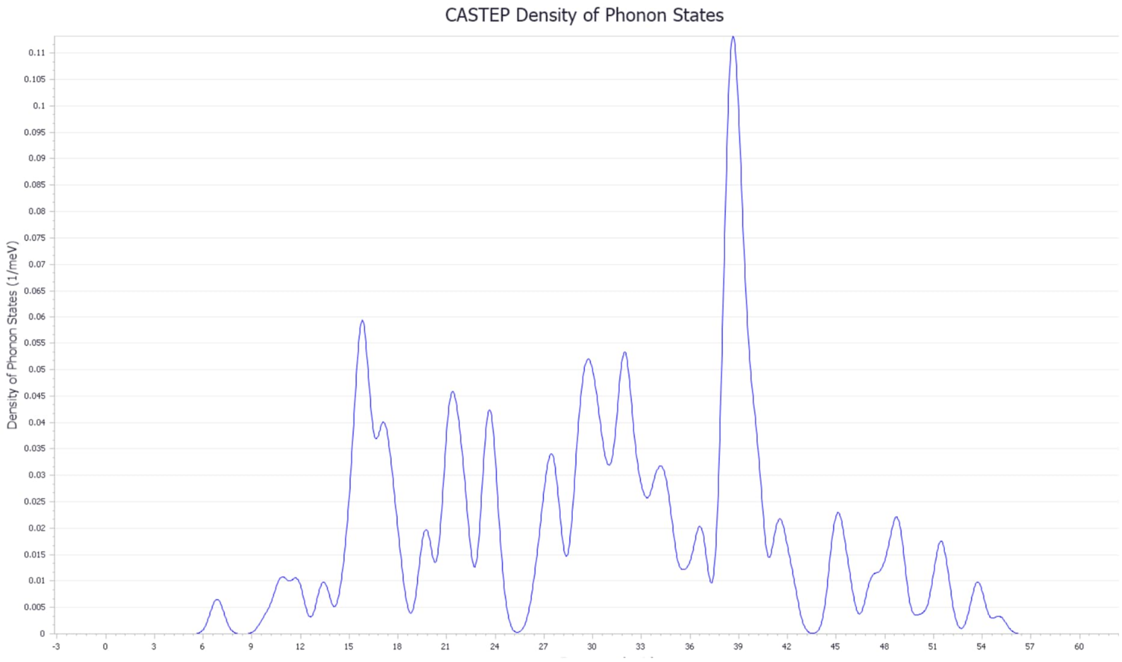

Figure S9. Total VDOS from the DFT calculation for Na_2O.

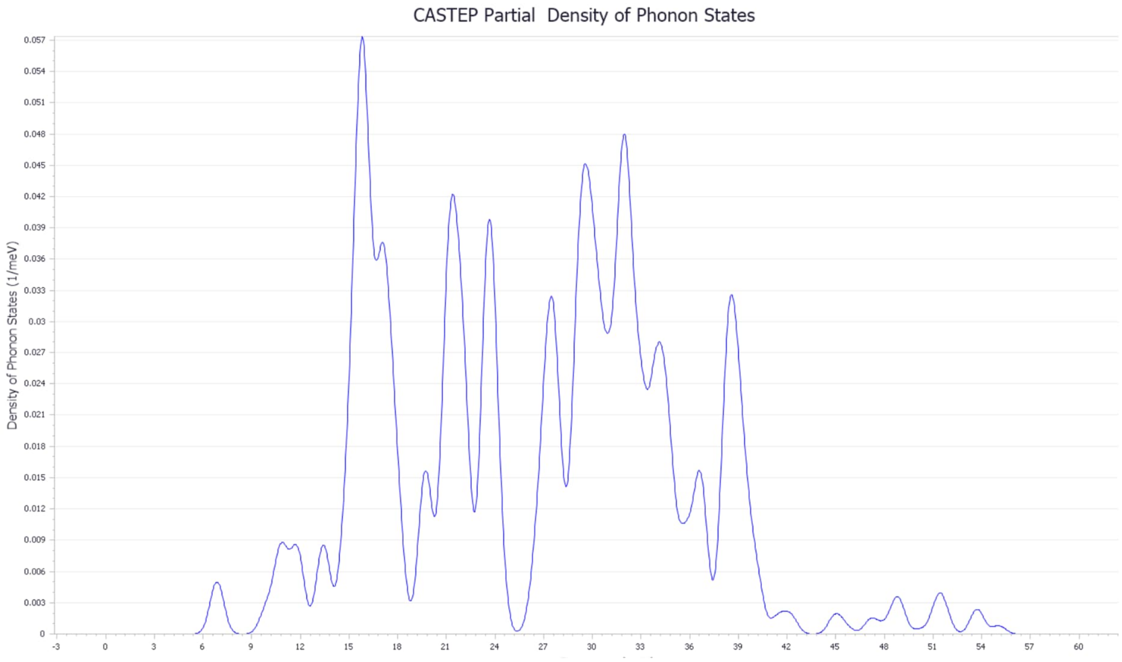

Figure S10. Partial Na-projected VDOS from the DFT calculation for Na_2O.

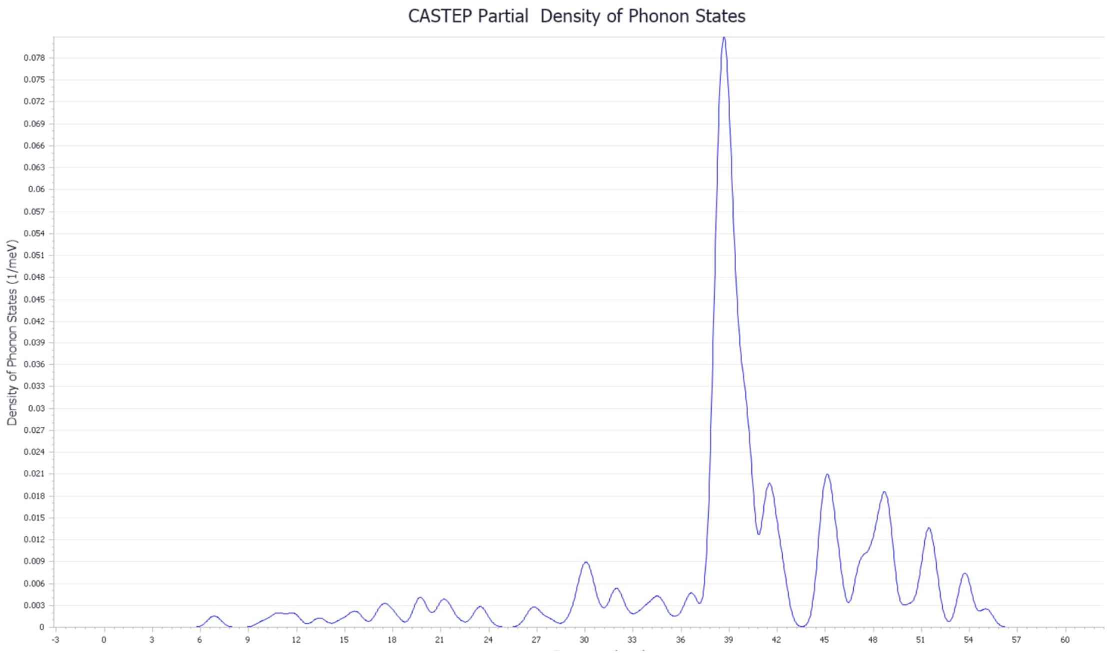

Figure S11. Partial O-projected VDOS from the DFT calculation for Na_2O.

3.3 P_2O_5

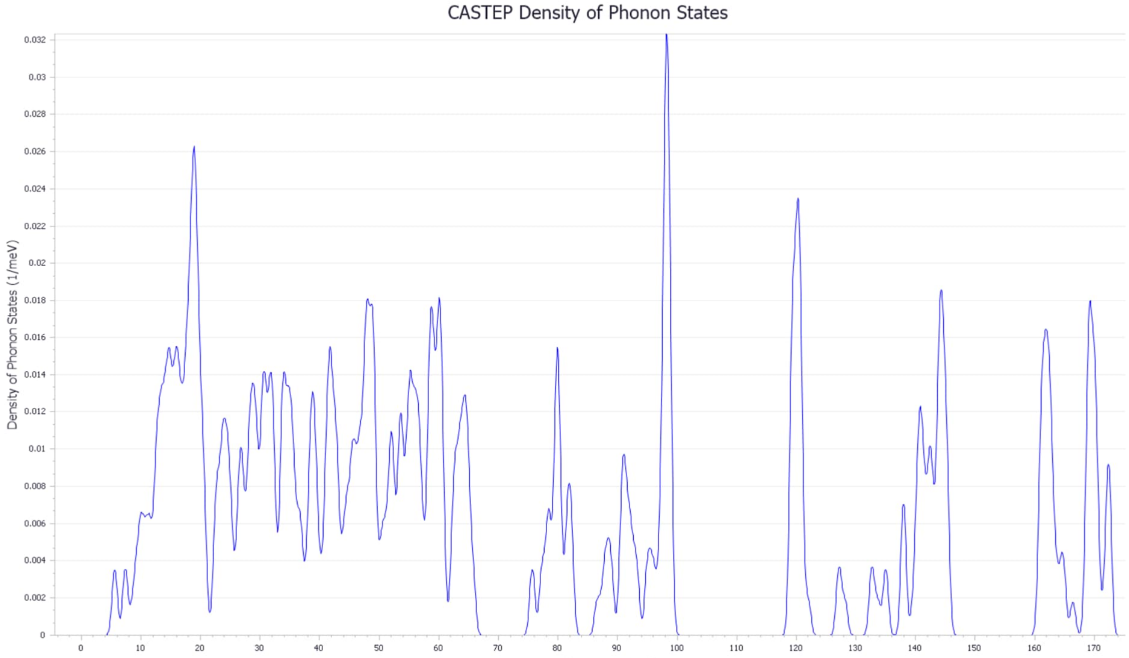

Figure S12. Total VDOS from the DFT calculation for P_2O_5.

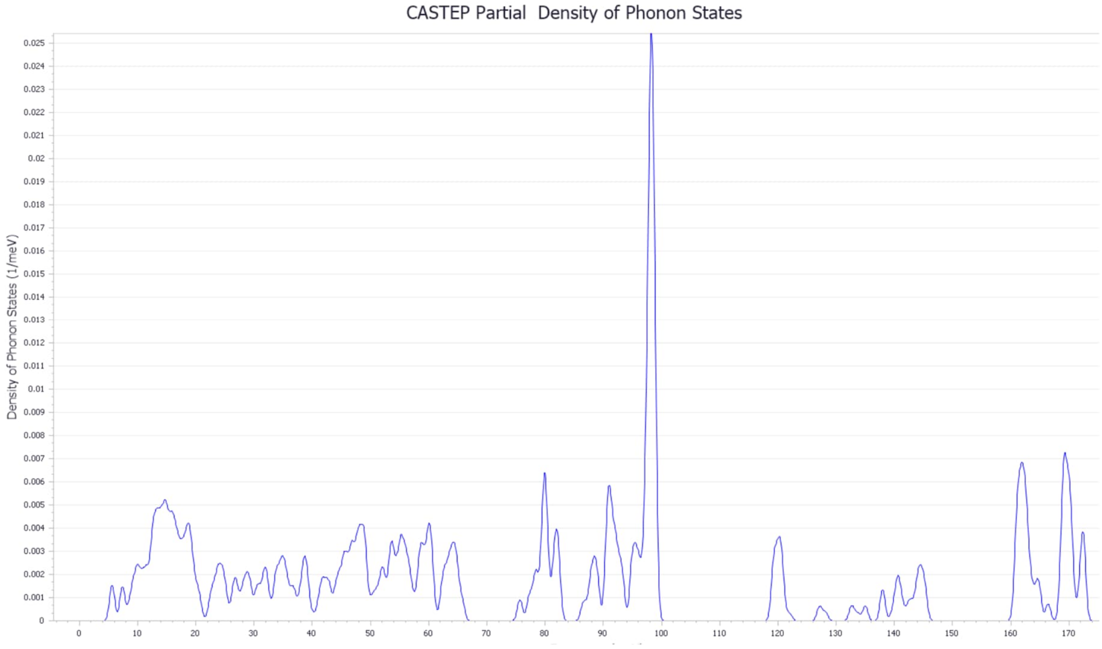

Figure S13. Partial P-projected VDOS from the DFT calculation for P_2O_5.

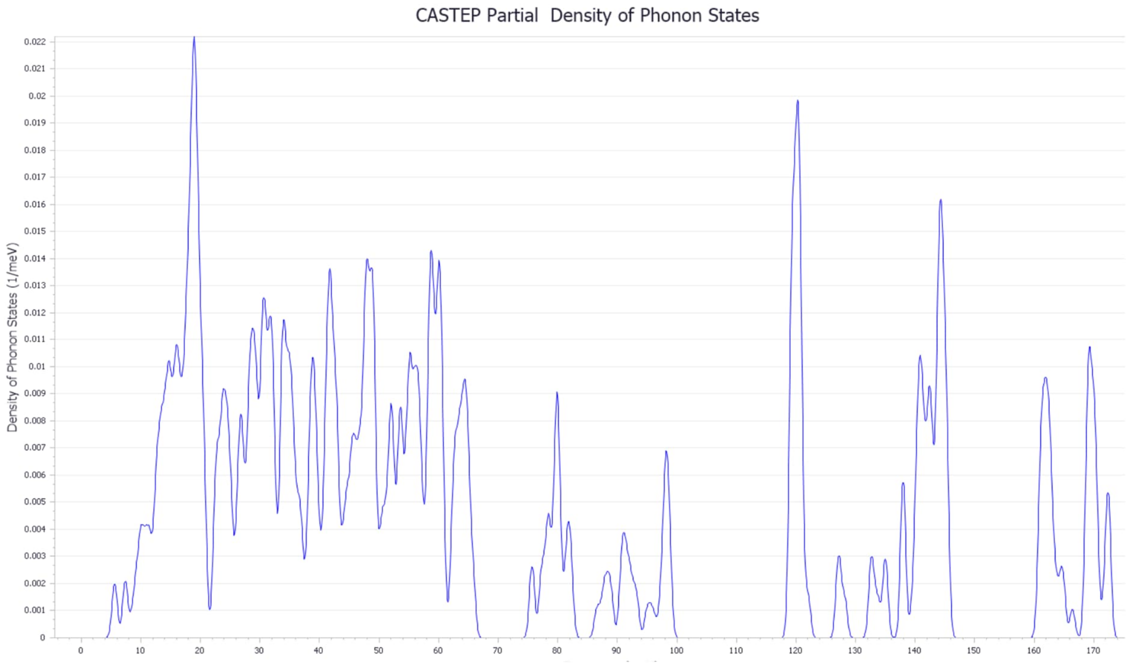

Figure S14. Partial O-projected VDOS from the DFT calculation for P_2O_5.

3.4 SiO_2

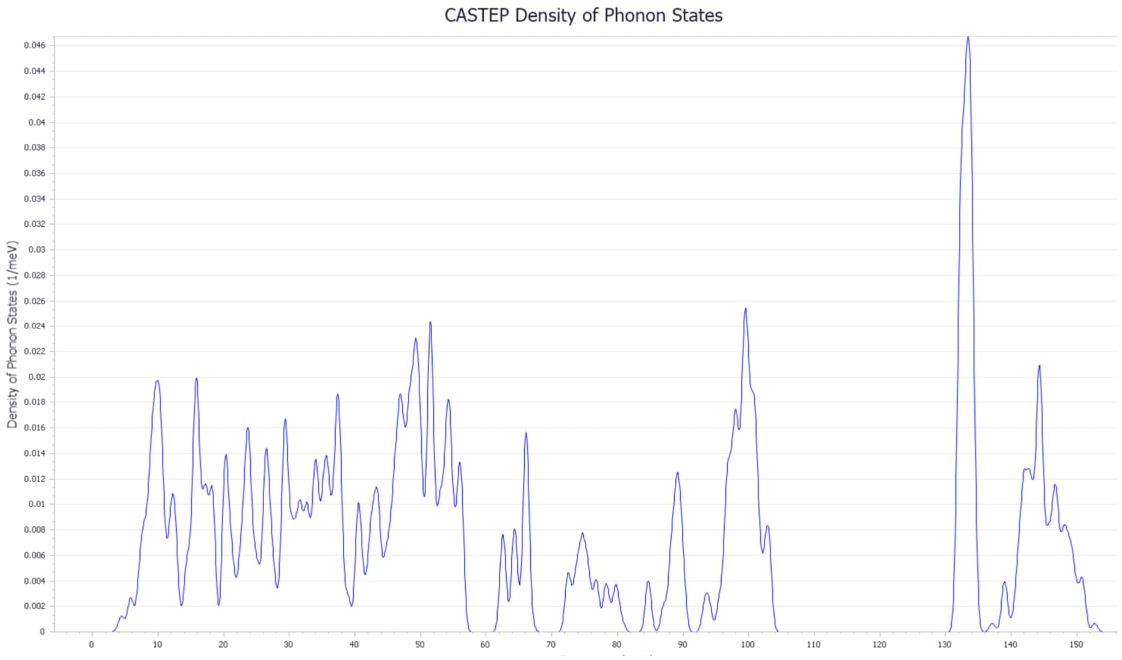

Figure S15. Total VDOS from the DFT calculation for SiO_2.

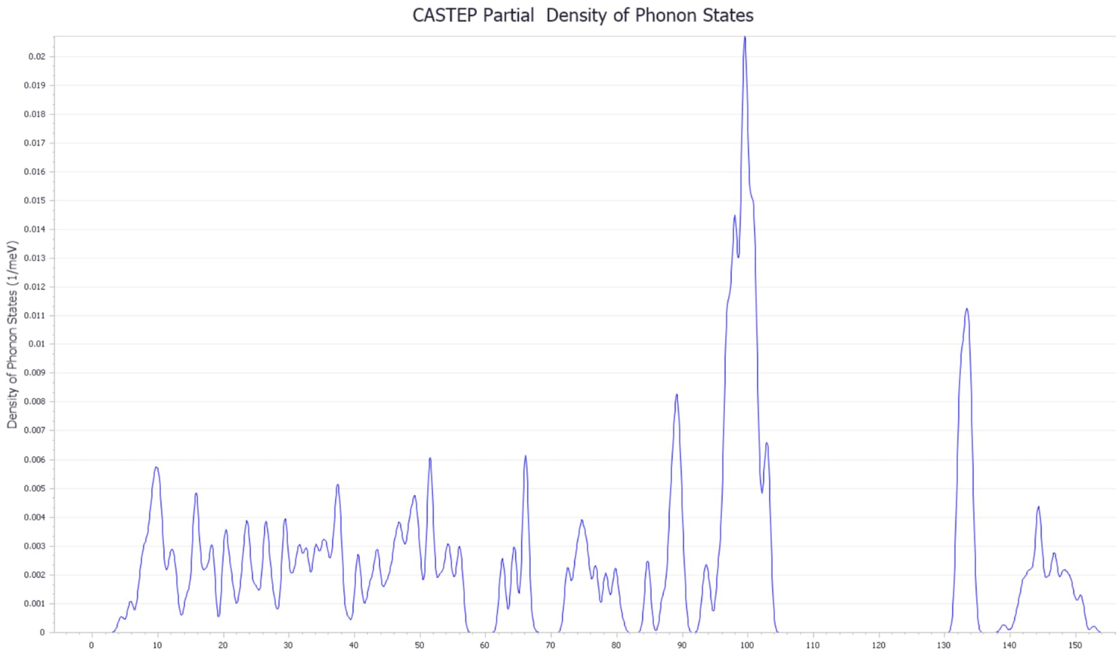

Figure S16. Partial Si-projected VDOS from the DFT calculation for SiO_2.

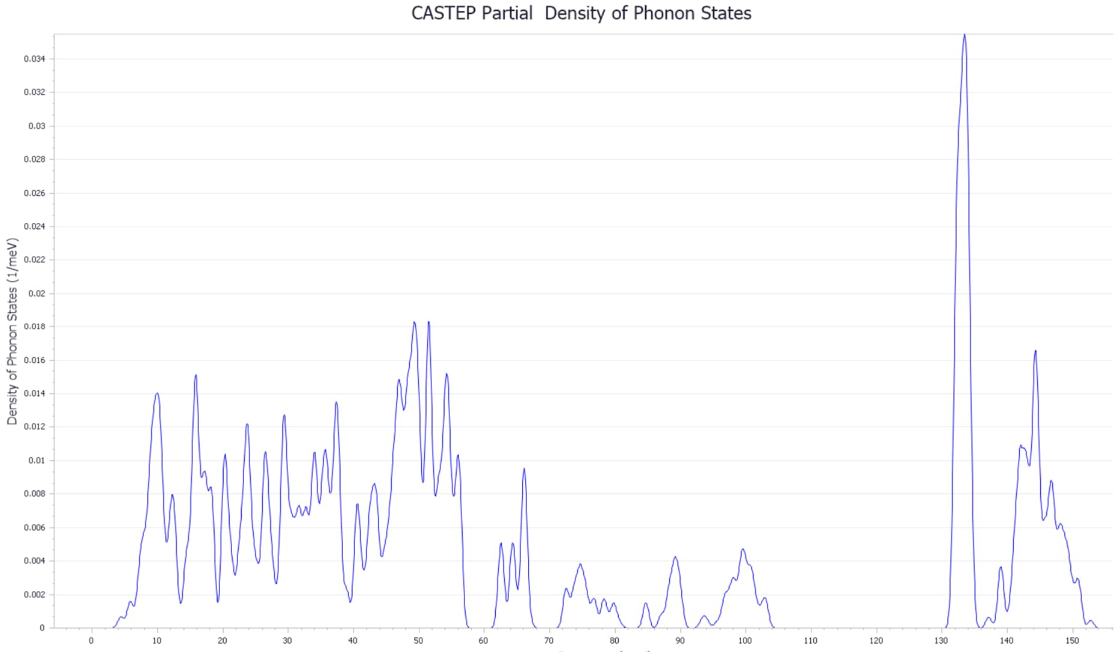

Figure S17. Partial O-projected VDOS from the DFT calculation for SiO_2.

3.5 SrO

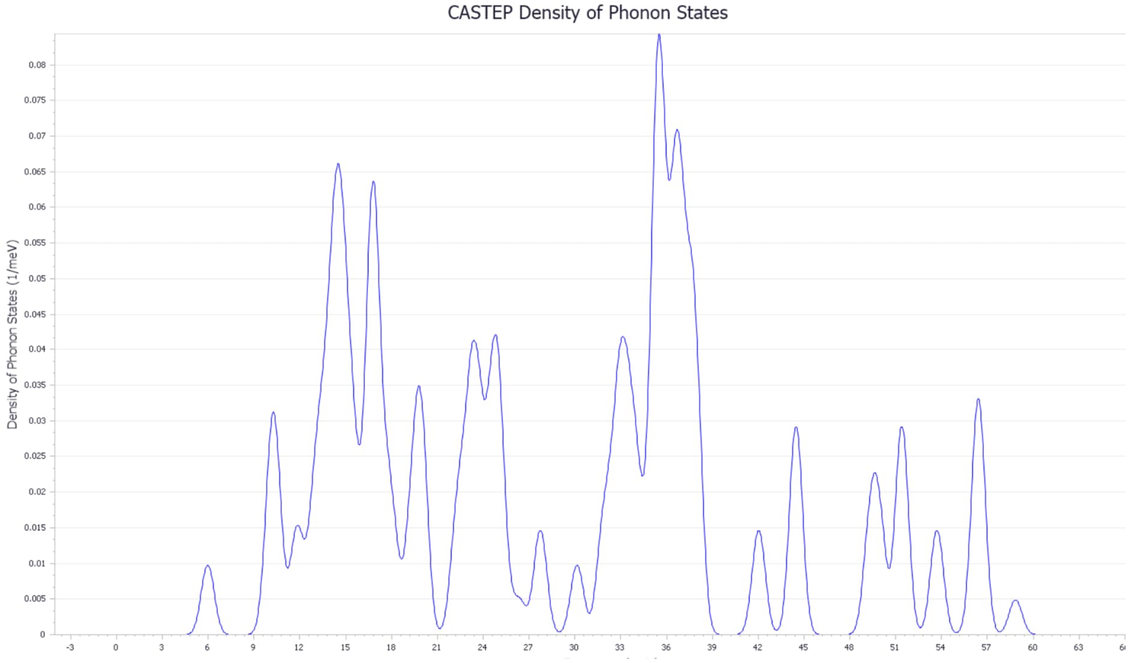

Figure S18. Total VDOS from the DFT calculation for SrO.

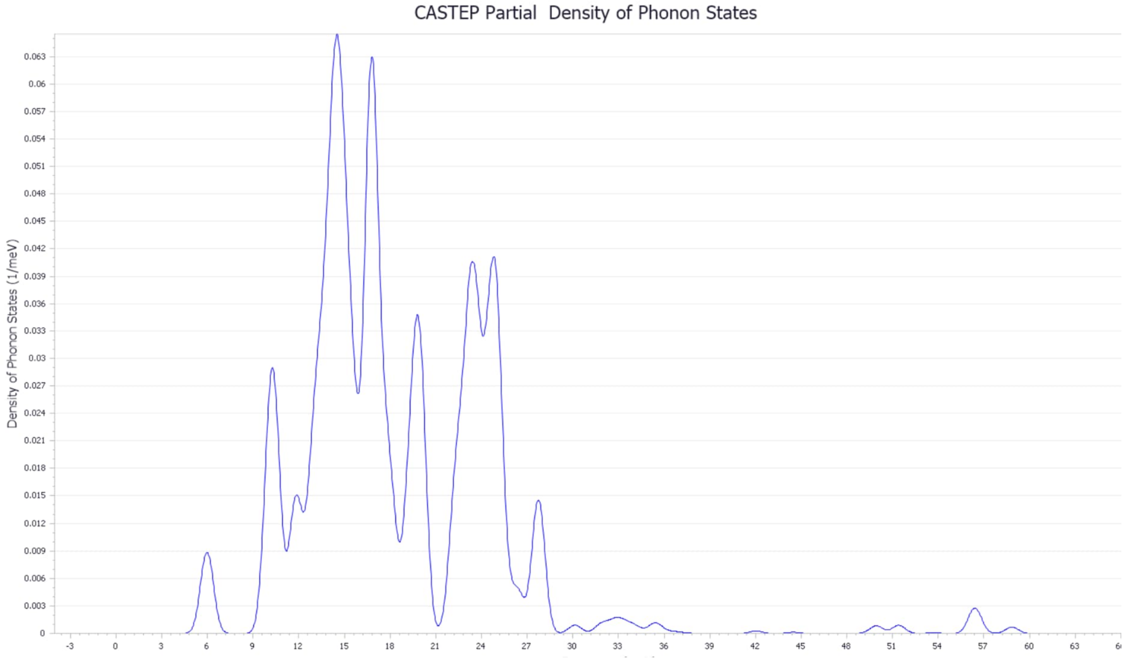

Figure S19. Partial Sr-projected VDOS from the DFT calculation for SrO.

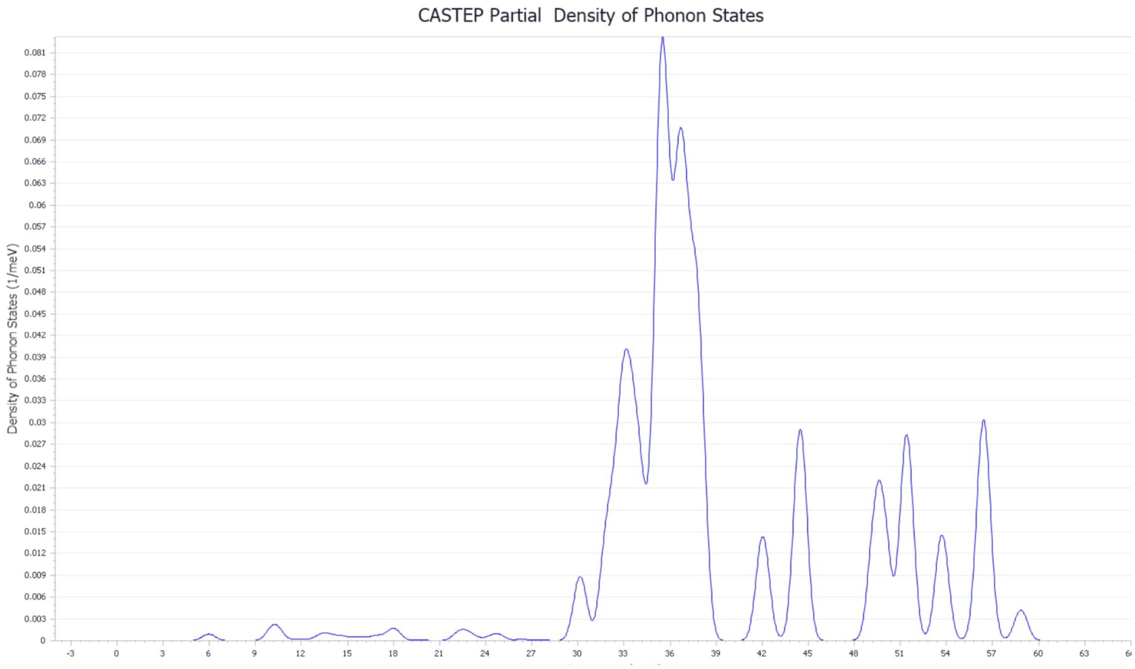

Figure S20. Partial O-projected VDOS from the DFT calculation for SrO.

4. Neutron Compton Scattering experiments on parent metal oxides

4.1 CaO

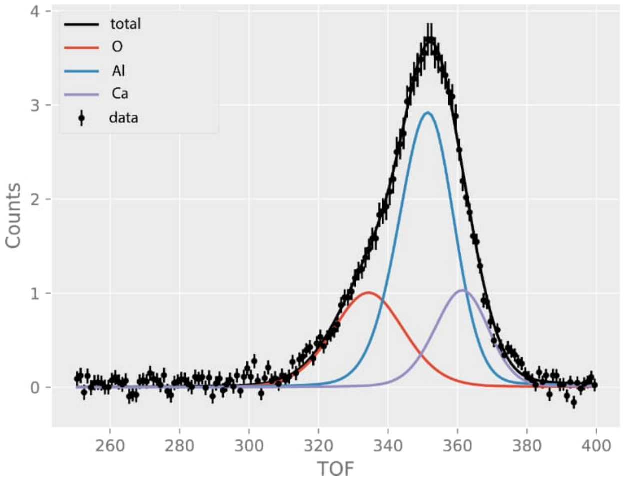

Figure S21. The NCS data of CaO. The sum of the neutron Compton backscattering spectra (black solid points and error bars) and the sum of the fits to the backscattering spectra recorded by individual detectors (black solid lines) for CaO. The sum of the fits of the recoil peaks of the oxygen (solid red line), aluminium (solid blue line), and calcium (solid violet line).

**Atom type**	**NMD width (Å^{-1})**	
	**experiment**	**DFT**
O	11.65 ± 0.28	11.07
Ca	16.65 ± 0.07	16.67

Table S11. Comparison of experimental and simulated values of the NMD widths for CaO.

4.2 Na_2O

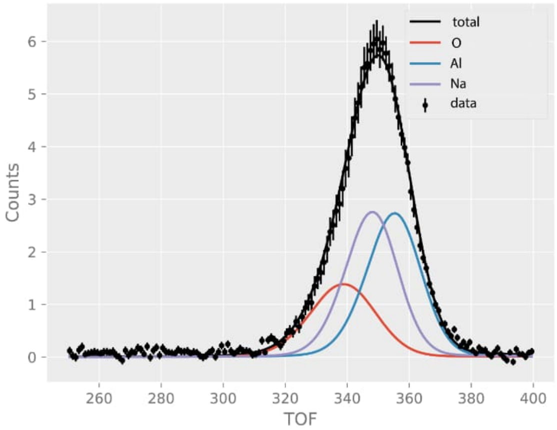

Figure S22. The NCS data of Na_2O. The sum of the neutron Compton backscattering spectra (black solid points and error bars) and the sum of the fits to the backscattering spectra recorded by individual detectors (black solid lines) for Na_2O. The sum of the fits of the recoil peaks of the oxygen (solid red line), aluminium (solid blue line), and sodium (solid violet line).

**Atom type**	**NMD width (Å^{-1})**	
	**experiment**	**DFT**
O	10.96±0.15	10.87
Na	11.93±0.07	12.48

Table S12. Comparison of experimental and simulated values of the NMD widths for Na_2O.

4.3 SrO

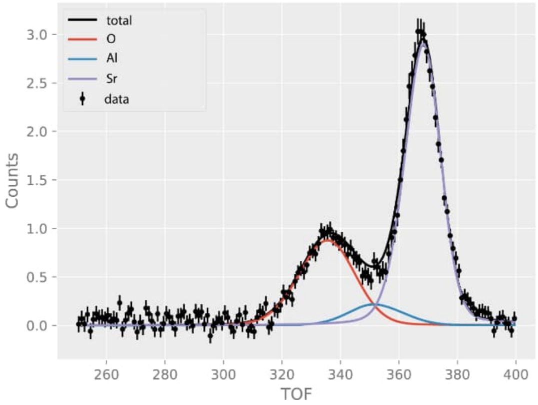

Figure S23. The NCS data of SrO. The sum of the neutron Compton backscattering spectra (black solid points and error bars) and the sum of the fits to the backscattering spectra recorded by individual detectors (black solid lines) for SrO. The sum of the fits of the recoil peaks of the oxygen (solid red line), aluminium (solid blue line), and strontium (solid violet line).

**Atom type**	**NMD width (Å^{-1})**	
	**experiment**	**DFT**
O	9.95±0.05	10.88
Sr	23.28±0.26	23.82

Table S13. Comparison of experimental and simulated values of the NMD widths for SrO.

4.4 SiO_2

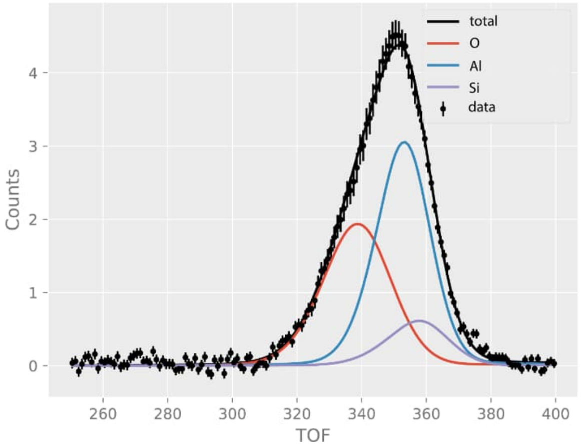

Figure S24. The NCS data of SiO_2. The sum of the neutron Compton backscattering spectra (black solid points and error bars) and the sum of the fits to the backscattering spectra recorded by individual detectors (black solid lines) for SiO_2. The sum of the fits of the recoil peaks of the oxygen (solid red line), aluminium (solid blue line), and silicon (solid violet line).

**Atom type**	**NMD width (Å^{-1})**	
	**experiment**	**DFT**
O	11.73±0.11	12.68
Si	17.10±0.39	17.19

Table S14. Comparison of experimental and simulated values of the NMD widths for SiO_2.

4.5 P_2O_5

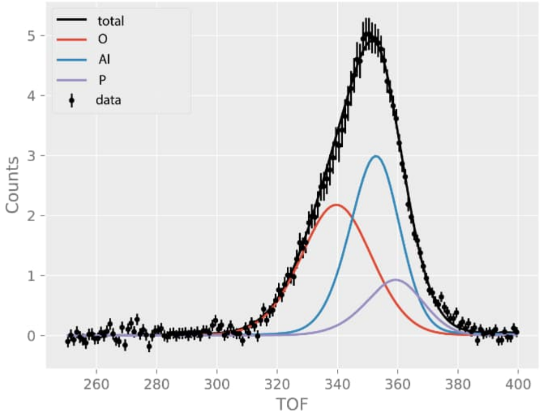

Figure S25. The NCS data of P_2O_5. The sum of the neutron Compton backscattering spectra (black solid points and error bars) and the sum of the fits to the backscattering spectra recorded by individual detectors (black solid lines) for P_2O_5. The sum of the fits of the recoil peaks of the oxygen (solid red line), aluminium (solid blue line), and phosphorus (solid violet line).

**Atom type**	**NMD width (Å^{-1})**	
	**experiment**	**DFT**
O	13.34±1.00	12.75
P	19.60±1.14	18.48

Table S15. Comparison of experimental and simulated values of the NMD widths for P_2O_5.
